# Cross-protective HCoV immunity reduces symptom development during SARS-CoV-2 infection

**DOI:** 10.1128/mbio.02722-23

**Published:** 2024-01-25

**Authors:** Irene A. Abela, Magdalena Schwarzmüller, Agne Ulyte, Thomas Radtke, Sarah R. Haile, Priska Ammann, Alessia Raineri, Sonja Rueegg, Selina Epp, Christoph Berger, Jürg Böni, Amapola Manrique, Annette Audigé, Michael Huber, Peter W. Schreiber, Thomas Scheier, Jan Fehr, Jacqueline Weber, Peter Rusert, Huldrych F. Günthard, Roger D. Kouyos, Milo A. Puhan, Susi Kriemler, Alexandra Trkola, Chloé Pasin

**Affiliations:** 1 Institute of Medical Virology, University of Zurich, Zurich, Switzerland; 2 Department of Infectious Diseases and Hospital Epidemiology, University Hospital Zurich, University of Zurich, Zurich, Switzerland; 3 Epidemiology, Biostatistics and Prevention Institute (EBPI), University of Zurich, Zurich, Switzerland; 4 University Children Hospital Zurich, Zurich, Switzerland; 5 Collegium Helveticum, Zurich, Switzerland; University of California, Davis, Davis, California, USA

**Keywords:** SARS-CoV-2, pre-exisiting immunity, cross-immunity, respiratory infection, HCoV, children

## Abstract

Numerous clinical parameters link to severe coronavirus disease 2019, but factors that prevent symptomatic disease remain unknown. We investigated the impact of severe acute respiratory syndrome coronavirus type 2 (SARS-CoV-2) and endemic human coronavirus (HCoV) antibody responses on symptoms in a longitudinal children cohort (*n* = 2,917) and a cross-sectional cohort including children and adults (*n* = 882), all first exposed to SARS-CoV-2 (March 2020 to March 2021) in Switzerland. Saliva (*n* = 4,993) and plasma (*n* = 7,486) antibody reactivity to the four HCoVs (subunit S1 [S1]) and SARS-CoV-2 (S1, receptor binding domain, subunit S2 [S2], nucleocapsid protein) was determined along with neutralizing activity against SARS-CoV-2 Wuhan, Alpha, Delta, and Omicron (BA.2) in a subset of individuals. Inferred recent SARS-CoV-2 infection was associated with a strong correlation between mucosal and systemic SARS-CoV-2 anti-spike responses. Individuals with pre-existing HCoV-S1 reactivity exhibited significantly higher antibody responses to SARS-CoV-2 in both plasma (IgG regression coefficients = 0.20, 95% CI = [0.09, 0.32], *P* < 0.001) and saliva (IgG regression coefficient = 0.60, 95% CI = [0.088, 1.11], *P* = 0.025). Saliva neutralization activity was modest but surprisingly broad, retaining activity against Wuhan (median NT50 = 32.0, 1Q–3Q = [16.4, 50.2]), Alpha (median NT50 = 34.9, 1Q–3Q = [26.0, 46.6]), and Delta (median NT50 = 28.0, 1Q–3Q = [19.9, 41.7]). In line with a rapid mucosal defense triggered by cross-reactive HCoV immunity, asymptomatic individuals presented with higher pre-existing HCoV-S1 activity in plasma (IgG HKU1, odds ratio [OR] = 0.53, 95% CI = [0.29,0.97], *P* = 0.038) and saliva (total HCoV, OR = 0.55, 95% CI = [0.33, 0.91], *P* = 0.019) and higher SARS-CoV-2 reactivity in saliva (IgG S2 fold change = 1.26, 95% CI = [1.03, 1.54], *P* = 0.030). By investigating the systemic and mucosal immune responses to SARS-CoV-2 and HCoVs in a population without prior exposure to SARS-CoV-2 or vaccination, we identified specific antibody reactivities associated with lack of symptom development.

**IMPORTANCE:**

Knowledge of the interplay between human coronavirus (HCoV) immunity and severe acute respiratory syndrome coronavirus type 2 (SARS-CoV-2) infection is critical to understanding the coexistence of current endemic coronaviruses and to building knowledge potential future zoonotic coronavirus transmissions. This study, which retrospectively analyzed a large cohort of individuals first exposed to SARS-CoV-2 in Switzerland in 2020–2021, revealed several key findings. Pre-existing HCoV immunity, particularly mucosal antibody responses, played a significant role in improving SARS-CoV-2 immune response upon infection and reducing symptoms development. Mucosal neutralizing activity against SARS-CoV-2, although low in magnitude, retained activity against SARS-CoV-2 variants underlining the importance of maintaining local mucosal immunity to SARS-CoV-2. While the cross-protective effect of HCoV immunity was not sufficient to block infection by SARS-CoV-2, the present study revealed a remarkable impact on limiting symptomatic disease. These findings support the feasibility of generating pan-protective coronavirus vaccines by inducing potent mucosal immune responses.

## INTRODUCTION

Infection with severe acute respiratory syndrome coronavirus type 2 (SARS-CoV-2) can lead to highly diverse manifestations ranging from asymptomatic infection, severe forms of coronavirus disease 2019 (COVID-19), to death and long COVID syndrome (1–7). While numerous clinical conditions have been associated with disease severity (1, 8–10) and an increasing number of risk factors are linked to long COVID syndrome (5, 6, 11), parameters that define asymptomatic and mild outcomes have not been ascertained (12, 13). The high prevalence of asymptomatic and mild infections early in the pandemic (1, 2, 14, 15), when the virus encountered a SARS-CoV-2-naive population, is particularly intriguing, underlining that genetic and/or pre-existing immune factors may exist that partially restrict SARS-CoV-2 infectivity. Definition of these parameters will be important to prepare for future zoonotic coronavirus transmissions to humans and to evolve effective cross-protecting coronavirus vaccines (16–18). Although SARS-CoV-2 is not closely related to endemic human coronaviruses (HCoVs), sequence homologies exist and give rise to both cross-reactive antibody and T cell responses (18–26). Indeed, research from others and us has highlighted that pre-existing immunity to HCoVs may limit disease severity either directly (27) or by supporting the development of SARS-CoV-2-specific humoral (22, 27–29) and cellular (21) responses. Cross-reactive HCoV T cell responses (21, 30) and cross-reactive HCoV antibodies (31, 32) have been postulated to underlie the milder presentation of COVID-19 in children. Despite growing evidence, the impact of HCoV immunity on SARS-CoV-2 infection remains to be determined as it has not been observed in all settings (29, 33, 34). The disparity may have several reasons. Cross-reactivity can be assumed to be less effective than the later developing specific immunity and may thus depend on a high anti-HCoV activity which may only be maintained for a short period after an HCoV infection. Similarly, the effects of different HCoVs may vary, and geographic and temporal shifts in HCoV prevalence (29, 35–37) may influence HCoV immunity to SARS-CoV-2 infection, necessitating tightly controlled study settings to unravel and confirm cross-talks. Binding and neutralizing SARS-CoV-2 antibody levels in plasma provide strong correlates of vaccine efficacy (38–41); however, localized mucosal antibodies present at the port of entry may provide the most rapid and effective measure in limiting virus acquisition (42–46). Indeed, monitoring salivary SARS-CoV-2 antibodies during acute and convalescent stages of COVID-19 (44, 47–49) and after vaccination (48, 50–52) has highlighted the magnitude of the antibody response in saliva but its impact on disease severity remains to be resolved. However, if and how HCoV-specific antibodies in saliva contribute to the control of SARS-CoV-2 infection has not been resolved. Unraveling the protective effects of HCoV immunity against SARS-CoV-2 infection is generally complex due to the known cross-reactivity and co-stimulation of SARS-CoV-2 and HCoV immune responses (22, 28, 53–55),

Here, we studied coronavirus antibody activity in plasma and saliva in the initial SARS-CoV-2 infection wave in 2020–2021 in Switzerland through periods of low and high SARS-CoV-2 prevalence in two independent cohorts, a longitudinal children cohort and a cross-sectional mixed adult-children cohort. This setting allowed us to pinpoint individuals who encountered SARS-CoV-2 for the first time and, through this to explore the role of saliva antibodies to HCoV and SARS-CoV-2 in protecting against SARS-CoV-2 infection and progression.

## RESULTS

### Temporal resolution of systemic and mucosal antibody responses to SARS-CoV-2

**Fig 1:**
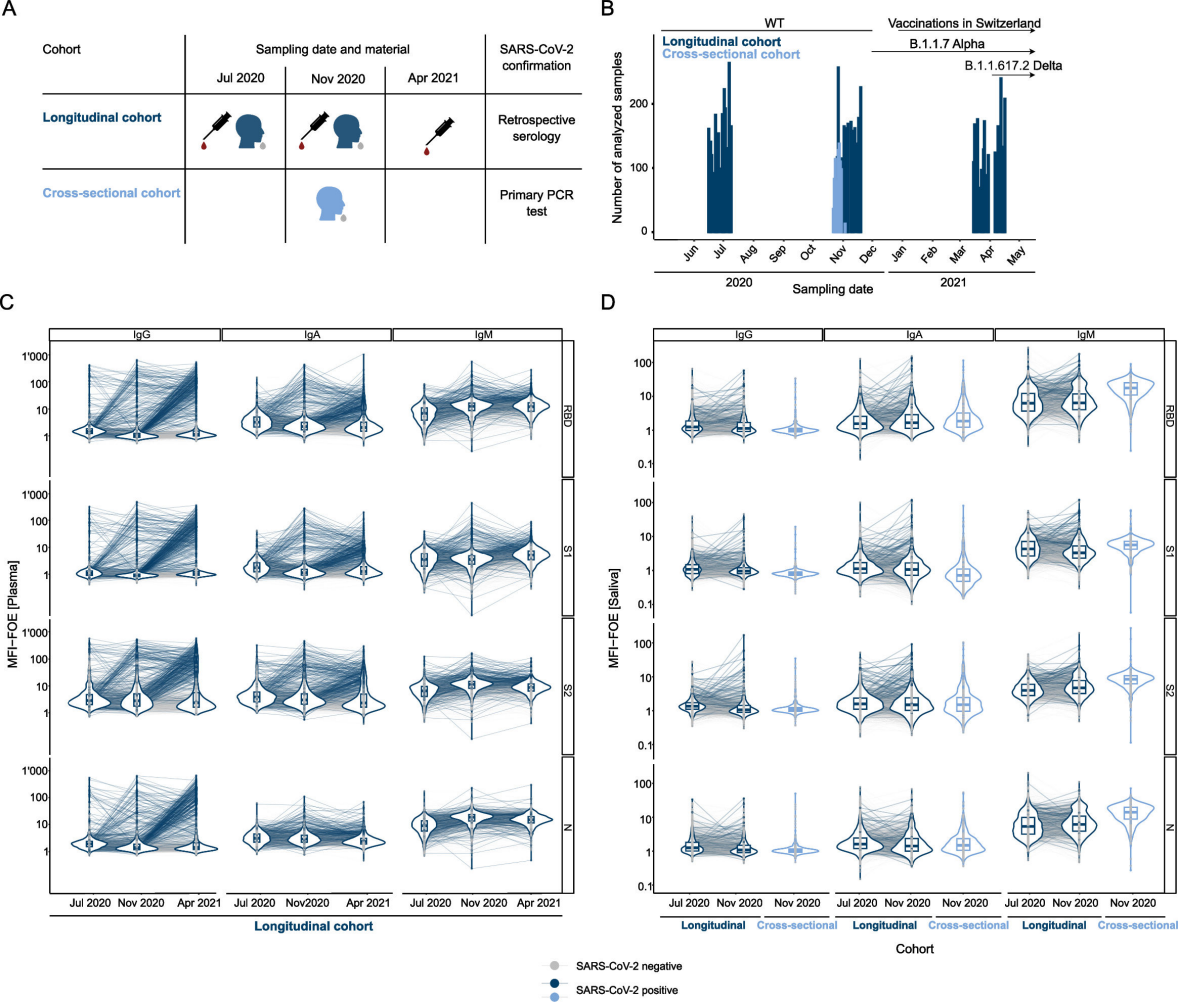
Multifactorial seroprofiling in a longitudinal cohort of children and a cross-sectional diagnostic cohort. (A) Schematic of serology sampling during three sampling rounds in the longitudinal cohort and one round in the cross-sectional cohort. Information is provided on the type of sampling for serology studies (plasma or saliva). (B) Sampling dates of the longitudinal (dark blue) and cross-sectional (light blue) cohorts. (C) Antibody measurements in the multiplex SARS-CoV-2 ABCORA in plasma in children from the longitudinal cohort with serology measurements at all three visit rounds (*n* = 1,967). Depicted are median fluorescence intensity (MFI) signals normalized for empty bead controls (fold over empty beads, MFI-FOE). Individuals in light gray stayed seronegative throughout the three visit rounds. Individuals in blue showed seroconversion. (D) Antibody measurements in the multiplex SARS-CoV-2 ABCORA in saliva on all children from the longitudinal cohort with serology measurements at the first two visit rounds (*n* = 2,806) and on individuals from the cross-sectional cohort (*n* = 882). SARS-CoV-2 positivity is determined by blood seropositivity in the longitudinal cohort (dark blue) or PCR positivity in the cross-sectional cohort (light blue). SARS-CoV-2 negative individuals (serology or PCR) are depicted in light gray.

We analyzed the HCoV and SARS-CoV-2 antibody titers using the multiplex ABCORA test (28, 56–63) in plasma and saliva of children and adults from two independent cohort studies conducted in Zurich, Switzerland, at the start of the pandemic and before the availability of SARS-CoV-2 vaccines for children and adults in Switzerland (March 2020 to March 2021) (56–59, 64) (see Table S1 for an outline of the study populations and the study design). The screen included concurrent saliva (*n* = 4,993) and plasma (*n* = 7,486) samples repeatedly collected during three different rounds (June–July 2020, October–November 2021, and March–April 2021) in a longitudinal seroprevalence children cohort, the Ciao Corona study (56–59) (referred to as longitudinal cohort hereafter; n_children_ = 2,917, aged 6–17 years; Fig. 1A through C; Fig. S1A; Table S2), in which SARS-CoV-2 was diagnosed retrospectively based on serological testing with the multiplex bead assay ABCORA (28). We further included saliva from adults and children collected in November 2020 through a cross-sectional test center cohort (64) (*n* = 882, aged 11–98 years; Fig. 1A, B, and D; Table S3). In this cross-sectional cohort, SARS-CoV-2 infection was diagnosed by polymerase chain reaction (PCR) in saliva and nasopharyngeal swabs.

Analysis of plasma (Fig. 1C) and saliva (Fig. 1D) in the longitudinal cohort revealed a lower but detectable SARS-CoV-2 antibody activity in saliva (Fig. S1B). Salivary antibody levels in the cross-sectional cohort (range of all median fluorescence intensities normalized for empty beads control [fold over empty beads] [MFI-FOE] = [−1.24, 2.44]) and the longitudinal cohort (range of all MFI-FOE = [−0.99, 2.44]) were in a similar range, suggesting comparability between the two cohorts.

**Fig 2:**
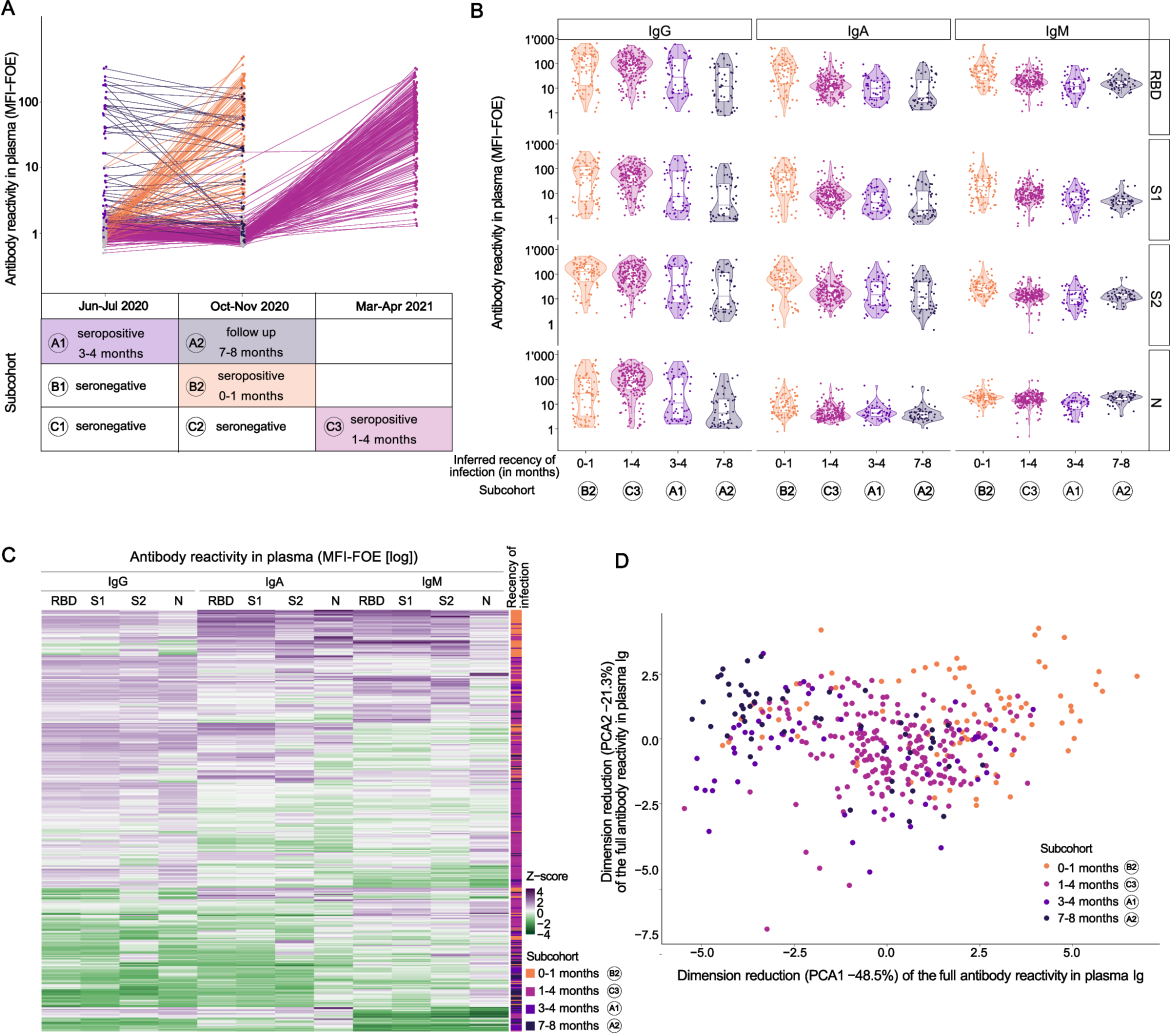
Inference of infection recency sub-cohorts based on multifactorial seroprofiling. (A) IgG receptor binding domain (RBD) MFI-FOE signals in sub-cohorts with different recency of infection, inferred using seropositivity assessment date and epidemic waves (see Fig. 1A). Sub-cohort A contains those seropositive in the first round (June–July 2020): measurements in June–July 2020 were realized 3–4 months after infection (A1, *n* = 56) and measurements in October–November 2020 were realized 7–8 months after infection (A2 = 52). Sub-cohort B contains those seronegative at the first round and seropositive at the second round. Measurements in October–November 2020 were realized within 1 month of infection (B2, *n* = 85). Sub-cohort C contains those seronegative at the first two rounds and seropositive at the last round (March–April 2021). Measurements in March–April 2021 were realized between 1 and 4 months post-infection (C3, *n* = 209). (B) Antibody measurements in the multiplex SARS-CoV-2 ABCORA in plasma of children in the different sub-cohorts. Depicted are MFI-FOE signals. (C) Heatmap representing Z-score of the MFI-FOE values of plasma samples in the different sub-cohorts. (D) Principal component analysis of plasma samples taken from the different sub-cohorts. (A, B, C, D) Sub-cohorts: B2: 0–1 month, orange; C3: 1–4 months, pink; A1: 3–4 months, light purple; A2: 7–8 months, dark purple.

Prospective sampling in the longitudinal cohort between June 2020 and April 2021 provided an ideal setting to resolve temporal associations of systemic and mucosal antibody responses. Because identification of SARS-CoV-2 infection in this cohort is based solely on retrospective detection of SARS-CoV-2 serum antibodies (56–59) and the exact time of infection is not known, we indirectly estimated the recency of infection by considering first detection of plasma SARS-CoV-2 antibodies in each individual and local epidemiological data, which was closely monitored by authorities, providing precise epidemiological information of SARS-CoV-2 prevalence in the canton of Zurich (Fig. 1B; Fig. S1A). We used this combined information to assign individuals from the longitudinal cohort to sub-cohorts depending on the inferred recency of infection (Fig. 2A). Sub-cohort A comprises individuals who were diagnosed as SARS-CoV-2 seropositive in the first sampling round; sub-cohort B comprises those diagnosed as SARS-CoV-2 negative in the first sampling round and positive in the second; sub-cohort C comprises those diagnosed as SARS-CoV-2 negative in the first and second sampling round and positive in the third (Fig. 2A; Fig. S2). For each of the sub-cohorts A–C, we analyzed specimens collected at the different sampling rounds (A1-2, B1-2, C1-3; Fig. 2A). Positive SARS-CoV-2 serology in June–July 2020, as seen in cohort A, most likely indicates infection during the first wave of the pandemic in Switzerland in March 2020, a period followed by a 6-week lockdown during which incidence rates declined rapidly (65). We thus inferred that sub-cohort A comprises individuals approximately 3–4 months post-infection (A1, *n* = 56). Follow-up of these individuals in the second sampling round in October–November 2020 (A2, *n* = 52) accordingly represents samples approximately 7–8 months post-infection. After two modest SARS-CoV-2 waves in spring and summer 2020, Switzerland experienced a high third wave in autumn 2020 (Fig. 1B; Fig. S1A) that overlapped with the second sampling round. Considering the low level of community transmission in-between first and second sampling timepoints in Switzerland, individuals that were first diagnosed seropositive at the second sampling (sub-cohort B) are thus most likely recent infections. We thus inferred that sub-cohort B at sampling round 2 (B2, *n* = 85) comprises individuals 0–1 months post-infection. Individuals who seroconverted by the third collection campaign in March–April 2021 were assigned to sub-cohort C (*n* = 209). Again, considering the local epidemiological data (Fig. 1B; Fig. S1A), seroconverters included in sub-cohort C were likely infected during the third wave that lasted from December 2020 to February 2021. We thus inferred that sub-cohort C, at sampling timepoint 3 (C3; *n* = 209), comprises samples collected 1–4 months post-infection.

This temporal resolution into different infection recency clusters was strongly reflected by distinctive characteristic IgG, IgA, and IgM patterns in plasma (Fig. 2B; Fig. S2) (28, 49). The early infection plasma samples at B2 showed the highest IgM and IgA reactivity to receptor binding domain (RBD) and subunit S1 (S1) (IgA RBD median [1Q, 3Q] in B2 = 1.72 [1.19, 2.01] [*n* = 85] versus 1.10 [0.85, 1.33], 1.00 [0.62, 1.29], and 0.60 [0.43, 1.34] in C3 [*n* = 209], A1 [*n* = 56], and A2 [*n* = 52], respectively; Fig. 2B and C; Fig. S2). IgG reactivity was highest in B2 and C3 (0–1 [*n* = 85] and 1–4 [*n* = 209] months of infection; IgG RBD median [1Q, 3Q] = 1.87 [1.11, 2.34] and 1.94 [1.59, 2.18] respectively) and declined thereafter in A1 and A2 (3–4 [*n* = 56] and 7–8 [*n* = 52] months of infection; IgG RBD median [1Q, 3Q] = 1.44 [0.79, 2.20] and 1.07 [0.46, 1.82] respectively), with a faster reduction in nucleocapsid protein (N) (fold change in A2 [*n* = 52] versus C3 [*n* = 209] = 0.42) than RBD reactivity (fold change = 0.60) as previously reported (28, 66). The analysis of the differential time-dependent clustering based on this seroprofiling confirmed the observations and showed a clear clustering of the assigned cohorts by their infection recency (Fig. 2C and D). From these analyses, we conclude that the assignment of SARS-CoV-2 infection recency in the longitudinal cohort is sufficiently accurate to allow combined analyses of saliva from the longitudinal cohort and saliva from the PCR-confirmed SARS-CoV-2 infections collected in the cross-sectional cohort at consecutive steps of the analysis.

### Pre-existing HCoV mucosal immunity shapes SARS-CoV-2 antibody response upon infection

**Fig 3:**
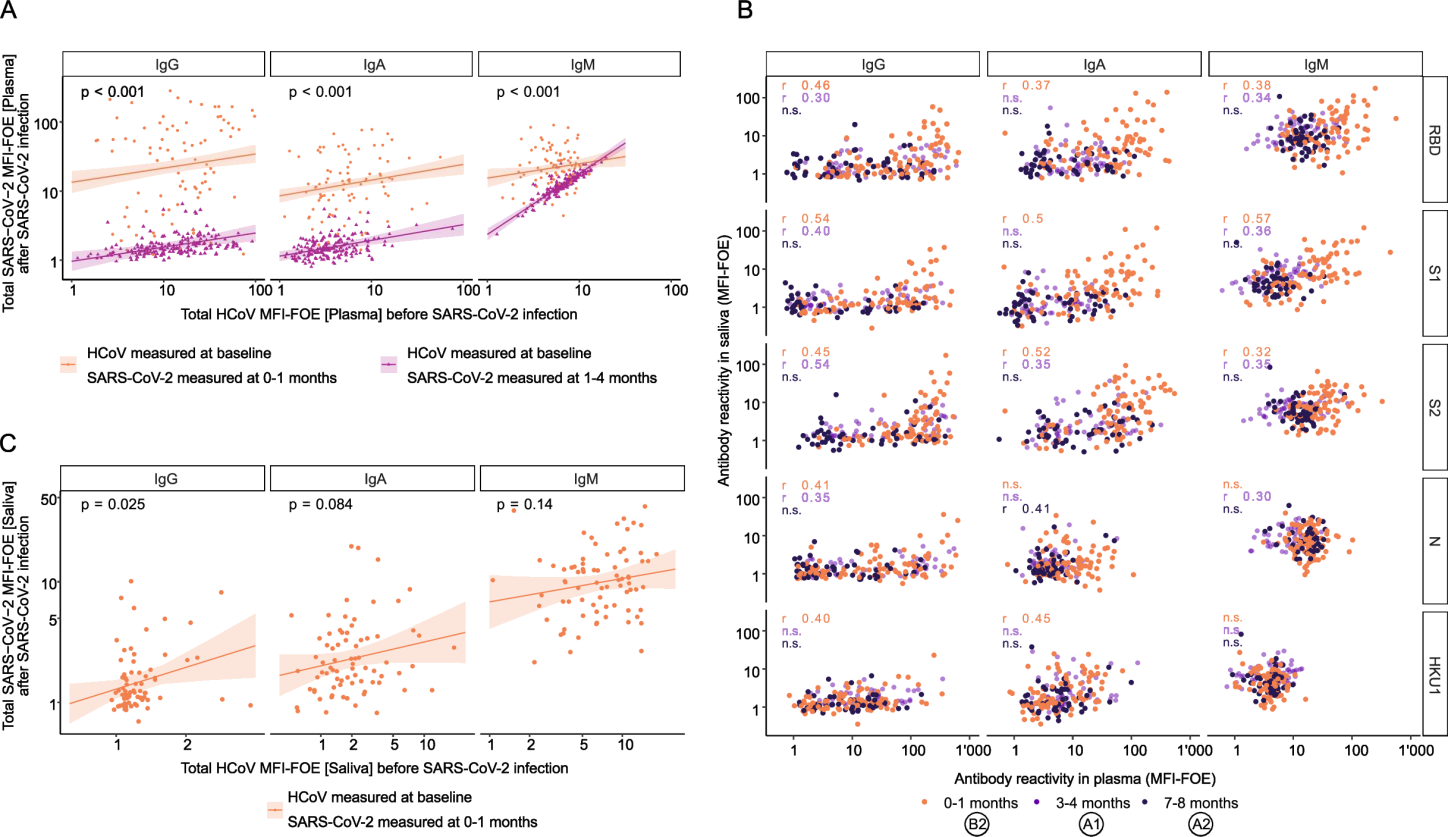
Pre-existing mucosal HCoV immunity shapes SARS-CoV-2 antibody response upon infection. (A) Linear regression analysis to define association between the total of SARS-CoV-2 antibody titers upon infection and the total of HCoV antibody titers before infection in plasma. Solid line indicates linear regression prediction (adjusted on age and sex) of individuals who tested positive at the second round (B2, orange, *n* = 85) and those who tested positive at the third round (C3, pink, *n* = 196). Shaded areas correspond to the 95% confidence intervals. (B) SARS-CoV-2 antibody response in saliva (MFI-FOE) against SARS-CoV-2 antibody response in plasma (MFI-FOE) from different infection recency sub-cohorts (B2: 0–1 month, orange, *n* = 85; A1: 3–4 months, light purple, *n* = 56; A2: 7–8 months, dark purple, *n* = 52). Spearman correlation coefficients are indicated. Non-significant coefficients (*P* > 0.05) are marked “n.s.” (C) Linear regression analysis to define association between the total SARS-CoV-2 antibody titers upon infection and the total HCoV antibody titers before infection in saliva. Solid line indicates linear regression prediction (adjusted on age and sex) of individuals who tested positive at the second round (B2, orange, *n* = 85). Shaded areas correspond to the 95% confidence intervals.

The extent to which pre-existing immunity to endemic HCoVs influences susceptibility and severity of SARS-CoV-2 infection has been controversially debated (22, 26–28, 67). Previous work by us and others strongly suggests a beneficial effect of HCoV immunity in reducing SARS-CoV-2 acquisition and disease severity (21, 22, 25, 27, 28), while other studies found no or little effect (33, 34). Here, we considered that the discrepancy in results may in part be due to a different relative timing of SARS-CoV-2 and HCoV infections in individual cohorts, as HCoV infections have a pronounced seasonality with the four HCoV strains fluctuating annually and geographically (29, 36, 37). We concluded that a pairwise analysis of pre-infection samples and early post-infection SARS-CoV-2 seropositive samples in a population from the same geographic region during the same time period is needed to provide a controlled framework for studying the impact of HCoV immunity on SARS-CoV-2 antibody responses. The above-defined sub-cohorts B and C comprised sampling at pre-infection (B1 and C2, respectively) and early after seroconversion (B2 and C3, respectively), providing specimens that fulfilled these criteria. We re-measured plasma of these cohorts with the ABCORA 5 test that records IgG, IgA, and IgM responses to S1 to all four HCoVs (HKU1, NL63, 229E, and OC43) next to SARS-CoV-2 antigens (28). Using a linear regression model adjusted for age and sex, we found that a higher pre-existing systemic HCoV plasma antibody response was significantly associated with higher systemic SARS-CoV-2-specific antibody response upon infection (*n* = 281, *P* < 0.001 for IgG, IgA, and IgM, regression coefficient = 0.20, 95% CI = [0.09, 0.32]; 0.23, 95% CI = [0.12, 0.33] for IgG and IgA, respectively; 0.88, 95% CI = [0.79, 0.98] and 0.21, 95% CI = [0.034, 0.38] for IgM in sub-cohorts C and B, respectively; Fig. 3A), corroborating the impact of pre-existing HCoV immunity on SARS-CoV-2 antibody development we noted previously (28).

Mucosal immunity is a critical first line of defense against respiratory viruses (45, 68–70). Analysis of saliva from sub-cohorts A and B for antibodies to SARS-CoV-2 and the four HCoVs showed increasing correlation of SARS-CoV-2-specific antibody-binding reactivities in serum and saliva with inferred recency of infection (B2, *n* = 85, 0–1 month inferred recency of infection, IgG S1 cor = 0.54, 95% CI = [0.37, 0.67], *P* < 0.001; A1, *n* = 56, 3–4 months inferred recency of infection, IgG S1 cor = 0.40, 95% CI = [0.15, 0.60], *P* = 0.002; A2, *n* = 52, 7–8 m inferred recency of infection, IgG S1 cor = −0.16, 95% CI = [−0.41, 0.12], *P* = 0.27; Fig. 3B). Monitoring saliva responses to SARS-CoV-2 and HCoV longitudinally in sub-cohort B (*n* = 76, pre-infection B1, seroconverted B2), we observed that high pre-existing mucosal HCoV antibody responses were associated with high mucosal SARS-CoV-2 antibody response upon infection (IgG regression coefficient = 0.60, 95% CI = [0.088, 1.11], *P* = 0.025; not significant for IgA and IgM with regression coefficients = 0.20, 95% CI = [−0.023, 0.42], *P* = 0.084 and 0.19, 95% CI = [−0.063, 0.45], *P* = 0.14, respectively; Fig. 3C). These patterns suggest that high IgG mucosal HCoV immunity favors stronger mucosal SARS-CoV-2 IgG response.

### Early induction of SARS-CoV-2 neutralizing activity in saliva

**Fig 4:**
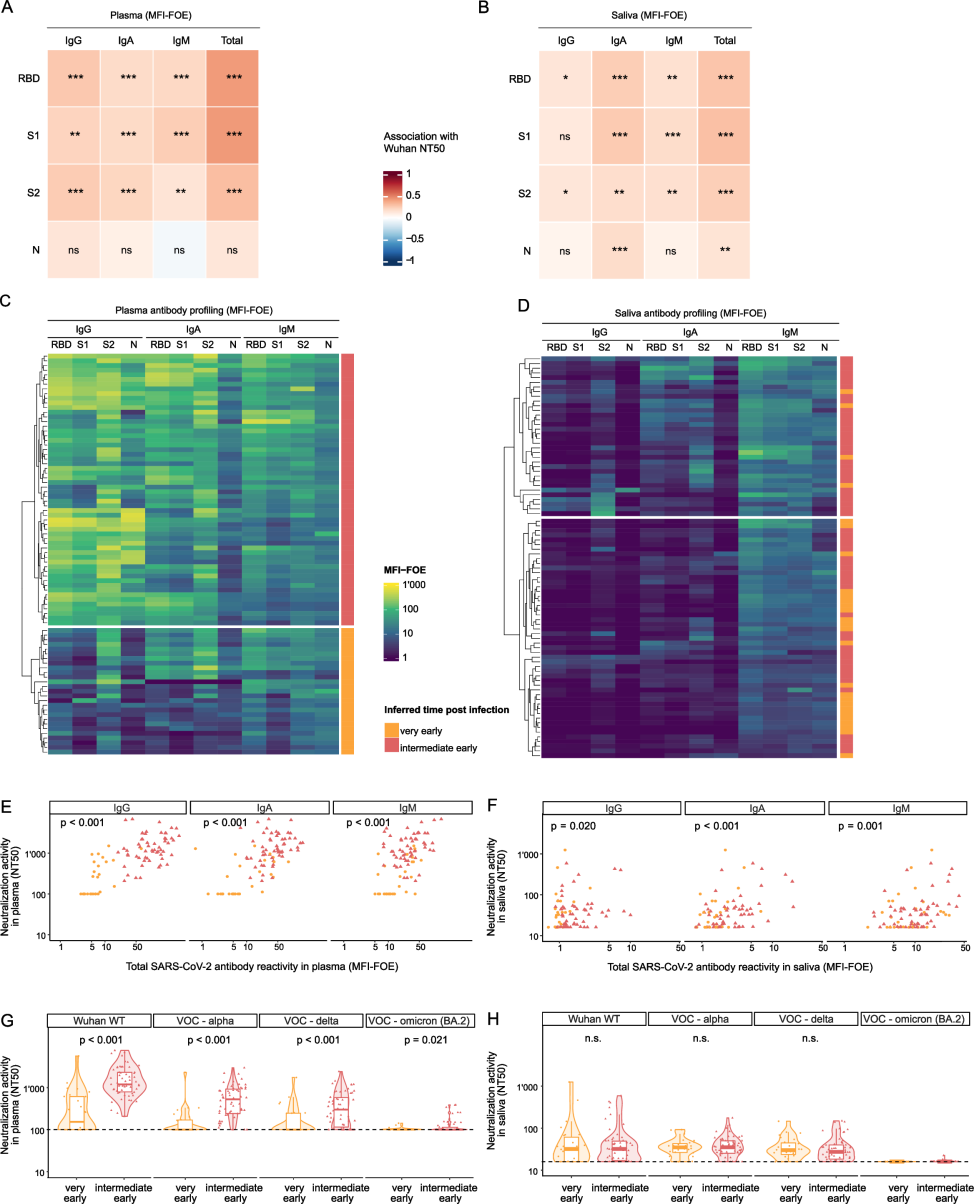
Mucosal antibodies display broad neutralizing activity. (A and B) Heatmap of association coefficients between neutralization titers (NT50) against Wuhan-Hu-1 pseudotype and IgG, IgA, and IgM SARS-CoV-2 antibody titers in plasma (*n* = 85) (A) and saliva (*n* = 85) (B) obtained with tobit-regression analyses adjusted on age and sex to define association. Levels of significance are indicated as follows: *P < 0.05, **P < 0.01, ***P < 0.001. (C and D) Heatmap representing MFI-FOE values of plasma (C) and saliva (D) samples of the cohort B2 (0–1 month, *n* = 85) and defining two subgroups of individuals: those very early after infection (no IgG response yet, orange, *n* = 27) and those intermediate early (IgG response detected, red, *n* = 58). (E and F) Association between neutralization titer (NT50) and total SARS-CoV-2 binding in plasma (E) and saliva (F) assessed in a tobit-regression model with all individuals grouped together (*n* = 85) and adjusted on age and sex. (G and H) Neutralization titers (NT50) against Wuhan-Hu-1 pseudotype, Alpha, Delta, and Omicron (BA.2) in plasma (G) and saliva (H) of individuals very early (orange circles, *n* = 27) and intermediate early (red triangles, *n* = 58) after infection. Dashed lines correspond to the limit of detection. *P*-values are obtained by comparing the two groups in tobit-regression models, adjusting for age and sex. Non-significant coefficients (*P* > 0.05) are marked “n.s.” No *P*-value was obtained for Omicron (BA.2) as most neutralization values are left-censored.

Rapid induction of neutralizing antibodies in the respiratory tract may allow suppression of SARS-CoV-2 infection preventing systemic spread and disease progression (38, 39). To explore the development of mucosal neutralizing antibodies in recent infection, we examined the neutralizing activity in saliva and plasma of the sub-cohort B early after seroconversion (B2, 0–1 month inferred recency of infection; Fig. 2A) against the Wuhan-Hu-1 strain, whose sub-lineages were prevalent when the sub-cohort B was infected (Fig. 1B; Fig. S1A). Neutralization activity against Wuhan-Hu-1 pseudovirus was frequent among B2 plasma samples (*n* = 73/85, 86%) and associated with spike IgG, IgA, and IgM but not N binding activities in tobit-regression models adjusted for age and sex (Fig. 4A). Notably, although low in magnitude, 77% of B2 saliva samples (*n* = 65/84) also neutralized Wuhan-Hu-1 pseudoviruses and this activity was most strongly linked with IgA responses followed by IgM and, to a lesser extent, by IgG (Fig. 4B).

As SARS-CoV-2 neutralization activity evolves gradually with prolonged affinity maturation of antibody responses even beyond virus clearance (71–74), plasma neutralization activity is commonly low early in primary infection (61, 75). To elucidate the dynamics of the neutralizing response in saliva and plasma during the earliest stages of disease, we used information from SARS-CoV-2 plasma antibody profiling to stratify sub-cohort B participants by infection recency at B2. Clustering based on plasma seroprofiles (Fig. 4C) identified two groups with distinct SARS-CoV-2 IgG reactivity. As plasma IgG responses require time to develop after infection (76), individuals with low plasma SARS-CoV-2 IgG were designated as very recent infection (*n* = 27), and individuals with a more developed SARS-CoV-2 IgG response were designated as intermediate early infection (*n* = 58). Notably, saliva seroprofiles did not reproduce this clustering (Fig. 4D), suggesting different dynamics of the antibody response in saliva.

In the identified very recent and intermediate early clusters, we found clear associations between neutralizing and spike-binding antibodies in plasma and saliva, suggesting that a low but rapidly established neutralizing response is active in the airway mucosa early in infection (Fig. 4E and F; Fig. S3A and B).

We further measured neutralization activity against Alpha, Delta, and Omicron (BA.2) in both B2 saliva and plasma samples (n_plasma_ = 85, n_saliva_ = 84; Fig. 4G and H) and compared it to neutralization activity against Wuhan-Hu-1. Neutralization activity in plasma showed the characteristic pattern: no or low neutralization activity against Wuhan-Hu-1 was detectable among individuals in the very recent infection cluster, but markedly increased in the intermediate early cluster (median Wuhan-Hu-1 NT50 [1Q, 3Q] in the very early = 153 [100, 616] versus 1,195 [793, 2,320] in the intermediate early, *P* < 0.001; Fig. 4G). Neutralization activity against the variants was significantly lower in both the very recent and intermediate early infection groups (*P* < 0.001 for Alpha, Delta, and Omicron BA.2 versus Wuhan-Hu-1) in a decreasing manner again following commonly seen trends (77–79).

Patterns of neutralization activity in saliva were strikingly different (Fig. 4H). Neutralizing activity against Wuhan-Hu-1, Alpha, and Delta in saliva (*n* = 84) was equivalent in the very recent and intermediate early infection groups (median Wuhan-Hu-1 NT50 [1Q, 3Q] in the very early = 31.9 [16, 61.6] versus 32.0 [17.0, 49.2] in the intermediate early, *P* = 0.78; median Alpha NT50 [1Q, 3Q] in the very early = 34.9 [26.4, 43.0] versus 35.1 [25.2, 50.0] in the intermediate early, *P* = 0.78; median Delta [1Q, 3Q] in the very early = 30.0 [23.7, 44.7] versus 27.3 [18.4, 39.9] in the intermediate early, *P* = 0.60), indicating a very rapid response that does not expand as infection becomes established. Equally striking, saliva neutralization against Alpha (median = 34.9, 1Q–3Q = [26.0, 46.6]) and Delta (median = 28.0, 1Q–3Q = [19.9, 41.7]) was maintained at almost the same level as against Wuhan-Hu-1 (median = 32.0, 1Q–3Q = [16.4, 50.2], *P* = 0.85 for Alpha, *P* = 0.26 for Delta) but was significantly decreased in Omicron BA.2 (median = 16.0, 1Q–3Q = [16.0, 16.0], *P* < 0.001). We conclude that salivary neutralizing activity is decoupled from the systemic response and is signified by a rapid induction of neutralizing activity that is maintained at a low level. As expected from the different dynamics, we found no (Wuhan-Hu-1 and Alpha) or only modest (Delta) correlation between neutralization in saliva and in plasma (Fig. S3C).

### Mucosal SARS-CoV-2 antibody response is associated with lower frequency of symptoms

**Fig 5:**
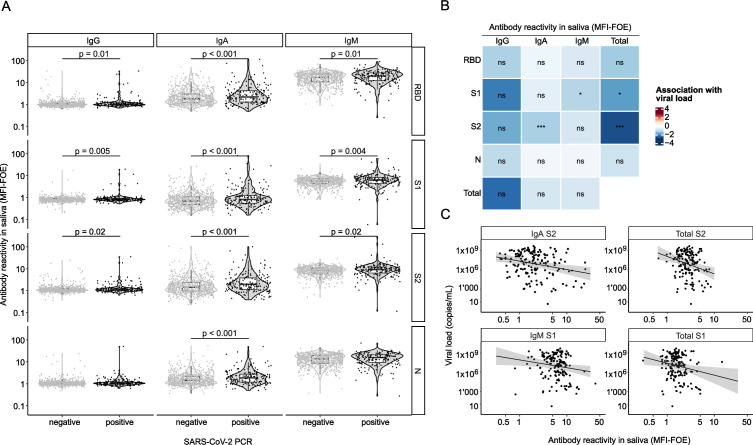
Mucosal immunity in the cross-sectional cohort. (A) Assessment of the multiplex SARS-CoV-2 ABCORA in saliva of PCR-positive (black, *n* = 177) and PCR-negative (gray, *n* = 705) individuals from the cross-sectional cohort. Differences were assessed using a linear regression adjusted on age and sex. (B) Association coefficient between SARS-CoV-2 antigen reactivity (RBD, S1, S2, N) based on MFI-LFOE values and viral load in PCR-positive individuals (*n* = 177) obtained using a linear regression adjusting on age and sex. Levels of significance are indicated as follows: ns, *P* > 0.05, *P < 0.05, **P < 0.01, ***P < 0.001. (C) Viral load (copies/mL) against IgA S2, IgM S1, total S2, and total S1 reactivities in saliva of PCR-positive individuals (*n* = 177). Solid line indicates linear regression prediction (adjusted on age and sex), and shaded areas correspond to the 95% confidence intervals.

We next used the cross-sectional cohort (*n* = 882; Fig. 1A and C) in which the presence of symptoms was recorded at the time of saliva sampling in addition to SARS-CoV-2 PCR data in saliva (64) to study the effect of saliva antibodies on symptoms. In this cohort, SARS-CoV-2 antibody levels in PCR-confirmed cases (*n* = 177) were low, as expected from an early stage after infection (Fig. 5A). We previously observed that SARS-CoV-2 antibodies in plasma are inversely related to SARS-CoV-2 virus loads in plasma and nasopharyngeal swabs (61) and sought to define whether salivary antibodies are associated with salivary viral load. Adjusting for age and sex, we found that four SARS-CoV-2 spike parameters, namely, IgA subunit S2 (S2), total Ig S2, IgM S1, and total Ig S1, were inversely associated with salivary viral load (fold change MFI-FOE = 0.27, 95% CI = [0.13, 0.57], *P* < 0.001, 0.11 [0.025,0.51], *P* < 0.001, 0.30 [0.11,0.81], *P* = 0.02, 0.15 [0.036, 0.66], *P* = 0.01, respectively; Fig. 5B and C).

**Fig 6:**
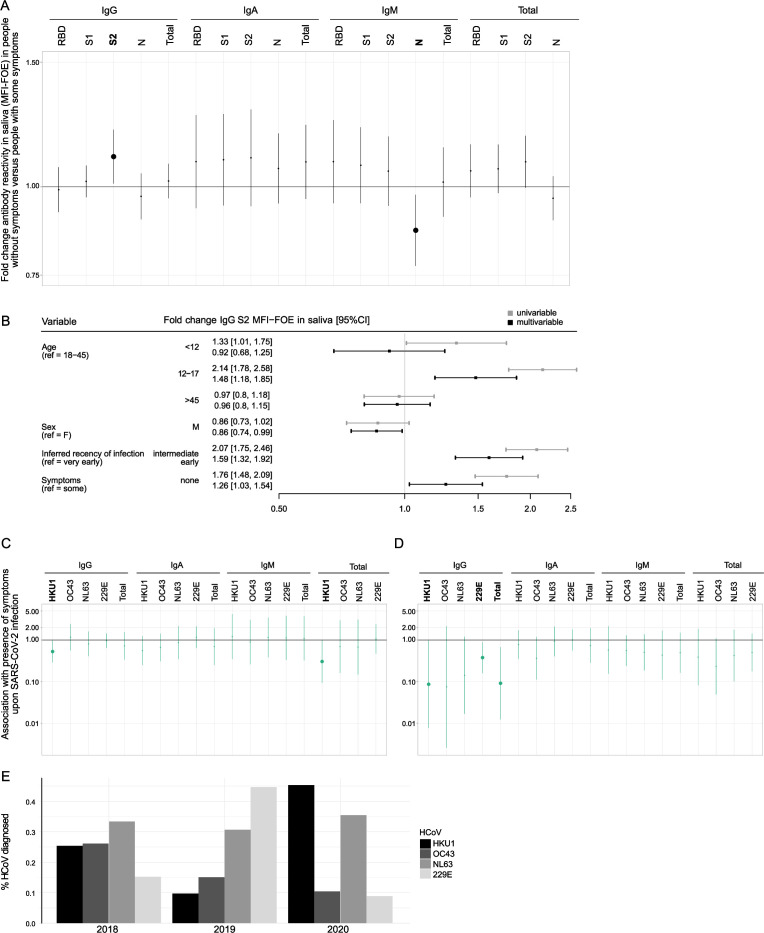
Cross-protective effect of HCoV immunity on symptoms. (A) Linear regression models (*n* = 219) adjusted on age, sex, and inferred recency of infection to assess the fold change in antibody reactivities in saliva (MFI-FOE) in individuals without symptoms versus individuals with symptoms. Binding activities with significant difference (*P* < 0.05) between those with and without symptoms are indicated in bold. Solid line indicates the fold change obtained from the linear regression model, and shaded areas correspond to the 95% confidence intervals. (B) Coefficient plot showing association of symptom development with mucosal IgG S2 antibody levels in univariable (gray) and multivariable (black) linear regression models adjusted on other covariables (age, sex, and inferred recency of infection). (C and D) Logistic regressions adjusted on age, sex, and inferred recency of infection (B2, 0–1 month or C3, 1–4 months) to assess association between symptom development upon SARS-CoV-2 infection and all pre-infection HCoV antibody titers in plasma (*n* = 215) (C) and saliva (*n* = 219) (D). Solid line indicates odds ratio estimation and shaded areas correspond to the 95% confidence intervals. Binding activities significantly associated with symptom development (*P* < 0.05) are indicated in bold. (E) Bar graph depicting prevalence of each HCoV among all HCoV cases diagnosed in 2018–2020 from the virus diagnostics unit at the University of Zurich.

We next used linear regression models adjusted for age, sex, and inferred recency of infection to systematically assess associations of SARS-CoV-2 binding activities with symptom development (total *n* = 219, including *n* = 64 from the longitudinal cohort and *n* = 155 from the cross-sectional cohort divided in very early, *n* = 162, intermediate early, *n* = 57). These analyses revealed that symptomatic individuals had higher plasma IgG (RBD, S1, N) responses than those who were asymptomatic in line with a higher exposure to viral antigen (Fig. S4A). In contrast, asymptomatic individuals had higher IgG S2 (fold change MFI-FOE = 1.26, 95% CI = [1.03, 1.54], *P* = 0.030) and lower IgM N (fold change MFI-FOE = 0.87, 95% CI = [0.77, 0.97], *P* = 0.017) mucosal antibodies than individuals who were symptomatic (Fig. 6A and B). While lower mucosal IgM N levels likely reflect lower exposure to antigen, the elevated mucosal IgG S2 level in asymptomatic individuals are highly intriguing and may indicate a direct protective activity.

### Pre-existing immunity to HCoVs reduces symptomatic SARS-CoV-2 infection

Local tissue responses and cross-reactive systemic humoral and cellular immunity to circulating HCoV have been suggested as factors influencing disease severity (21, 28, 30). To explore the impact of pre-existing HCoV immunity on symptom development, we utilized the longitudinal sub-cohorts B and C and measured HCoV antibody reactivity at their last negative timepoint before seroconversion in both plasma (total, *n* = 215: B1, *n* = 64, and C2, *n* = 151; Fig. 2A) and saliva (total, *n* = 219: B1, *n* = 58, and C2, *n* = 161; Fig. 2A). Controlling for age and sex, we found that individuals with high pre-existing plasma HKU1 IgG immunity reported significantly less symptoms following SARS-CoV-2 infection (*n* = 215, odds ratio [OR] = 0.53, 95% CI = [0.29, 0.97], *P* = 0.038; Fig. 6C; Fig. S4B). This was matched in the mucosal compartment, where we found that higher pre-infection levels in saliva for IgG HKU1, IgG 229E, and total HCoV-S1 IgG were associated with less frequent symptom development upon infection (*n* = 219, OR = 0.087, 95% CI = [0.0077,0 .9], *P* = 0.047, OR = 0.38, 95% CI = [0.16, 0.89], *P* = 0.027, and OR = 0.55, 95% CI = [0.33, 0.91], *P* = 0.019 respectively; Fig. 6D; Fig. S4C). A similar trend was observed for IgG NL63 levels (OR = 0.14, 95% CI = [0.017, 1.20], *P* = 0.073). Notably, these patterns reflected the test positivity rate of HCoV strains observed at our local virus diagnostics unit at the University of Zurich in the 2 years preceding the pandemic. Among specimens sent for testing with a respiratory virus panel in 2019 and early 2020, 229E infections were most prevalent in 2019, followed by higher rates of HKU1 and NL63 cases in 2020 (Fig. 6E). Collectively, these observations suggest that a recent exposure to 229E in 2019 or HKU1 in 2020 installed a cross-protective mucosal and systemic HCoV immune response in the investigated SARS-CoV-2 cohorts.

**Fig 7:**
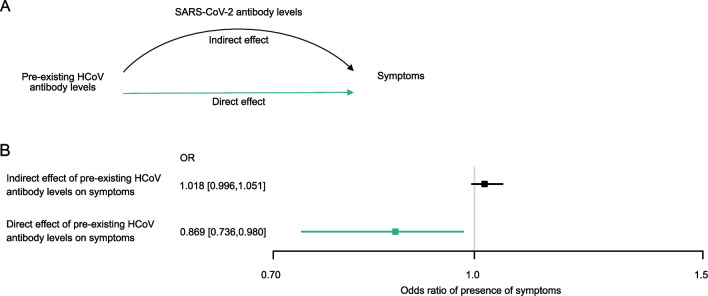
Cross-protective HCoV immunity has a direct impact on symptom development independent of the effect of SARS-CoV-2 antibody response. (A) Schematic depicting of mediation analysis investigating the direct effect of pre-existing HCoV antibody response in plasma on symptoms and its mediated effect through SARS-CoV-2 plasma antibody response upon infection. (B) Coefficients from the mediation analysis (*n* = 219), estimating the direct effect of pre-existing HCoV antibody response in plasma, and the indirect effect through SARS-CoV-2 plasma antibody response.

To explore whether the effect of pre-existing HCoV immunity on symptoms is only due to its role in enhancing the SARS-CoV-2 immune response upon infection, or whether additional mechanisms (potentially including the direct role of HCoV antibodies on viral clearance) are at play, we used the framework of mediation analysis in plasma (total, *n* = 226: B1–B2, *n* = 64, C2–C3, *n* = 162) and saliva (B1–B2, *n* = 58) (Fig. 7A). Owing to the small sample size, the analysis of saliva was not conclusive. However, intriguingly, we found that individuals with higher pre-infection plasma IgG HKU1-S1 levels are less likely to report symptoms [*n* = 226, direct effect OR = 0.87, 95% CI = [0.74, 0.98], *P* = 0.024; Fig. 7B). In contrast, SARS-CoV-2 plasma antibody titers (total IgG) observed upon infection did not mediate that effect (*P* = 0.15) (Fig. 7B). Overall, these observations suggest that cross-protective HCoV immunity has at least in part a direct impact on symptom development upon the first acquisition of SARS-CoV-2 infection independently of specific SARS-CoV-2 antibodies.

## DISCUSSION

Determining the impact of specific and cross-reactive HCoV immunity on SARS-CoV-2 infection remains of great interest for understanding the interplay of current endemic viruses and providing insight for future zoonotic transmission of coronaviruses (24–26, 80, 81). Here, we studied the effect of HCoV antibody responses in a population first encountering SARS-CoV-2, thereby restricting the layers of immune cross-stimulation to the initial boost in SARS-CoV-2-specific antibody activity.

In line with mucosal antibody responses providing a strong defense barrier against other respiratory infections (45, 68–70, 82–84), we observed a strong impact of HCoV- and SARS-CoV-2-specific responses in saliva on early SARS-CoV-2 infection. Pre-existing HCoV antibodies enhanced the development of SARS-CoV-2-specific activity and were linked with reduced disease severity (28). As evident from the diagnostic cross-sectional cohort, SARS-CoV-2 antibody responses in saliva were, although low in magnitude, present early after infection and inversely correlated with viral load levels in the nasopharynx, indicating a pronounced capacity to restrict local virus replication. Individuals with high SARS-CoV-2-specific IgG S2 antibody levels in saliva less frequently developed symptoms, providing further evidence that S2-specific B and T cell responses contribute to protection (54, 85–88). Neutralizing activity in saliva was low but surprisingly maintained some breadth against Alpha and Delta and was most strongly linked to IgA spike activity. Taken together, these findings underscore the potential of mucosal immunity to locally reduce the spread of SARS-CoV-2 infection and thereby limit the development of symptoms. As with systemic responses, maintaining high levels of mucosal SARS-CoV-2 antibody activity after vaccination is challenging and, to current knowledge, levels decline even after multiple vaccinations (89–91). Investing in novel vaccine strategies specifically designed to induce long-lasting mucosal responses is therefore warranted (92–97).

Cross-protection conferred by immunity to endemic HCoVs, as we report here for high pre-existing mucosal HCoV antibody reactivity, has been observed by several (21–23, 28, 98–100) but not all studies (33, 34). Protective immunity to HCoV is not long-lived (101–103). Based on our observations, similar dynamics are likely to apply to cross-reactive HCoV responses. The effect may be more pronounced if HCoV exposure occurred in a window of 1 to 2 years before a first infection with SARS-CoV-2, consistent with the relatively rapid waning of HCoV immunity also estimated to occur between 1 and 2 years (104, 105). The relative timing of SARS-CoV-2 and HCoV infection and the timepoints at which the respective antibody levels are measured may lead to substantially different outcomes, explaining the partially conflicting observations reported in the literature. Time-controlled comparisons, as conducted in the present study, taking into account the infection waves of both endemic HCoVs and SARS-CoV-2 and the relative temporal distances of the sampling times to them, are therefore critical to resolve HCoV cross-protective effects.

HCoV antibody responses that we measure here may directly act against SARS-CoV-2 or constitute a surrogate measure for other protective immune responses such as cytotoxic T cells (21, 24, 25). Next to a direct effect of cross-reactive antibodies on SARS-CoV-2 (22, 25, 28, 106), cross-reactive antibodies may mature to SARS-CoV-2 specificity (53, 55) and cross-reactive helper T cells may promote early SARS-CoV-2 antibody induction, consistent with the observation that high HCoV antibody responses promote higher SARS-CoV-2 antibody responses upon infection (22, 25, 28, 106).

The present study represents a *post hoc* analysis of samples collected in the frame of two unrelated SARS-CoV-2 studies, neither of which was designed to experimentally dissect the causality of the protective activity of HCoV antibodies on reducing symptomatic SARS-CoV-2 infection. The analysis of self-reported symptoms, especially in children, should be viewed with caution. Indeed, the longitudinal Ciao Corona study, despite closely monitoring flu-like symptoms with bi-monthly questionnaires in participating children, observed no clear correlation between SARS-CoV-2 seropositivity and the reported mild symptoms in the full Ciao Corona cohort (56–59) . Here, we used the same data but focused only on participants who turned SARS-CoV-2-positive during the observation period (sub-cohorts A, B, C, total *N* = 350) and for whom symptoms and HCoV data were also recorded. Individuals with available pre- and post-SARS-CoV-2 timepoints of plasma (*n* = 226) and saliva (*n* = 58), symptom information, and HCoV antibody measurement were subjected into a mediation analysis (Fig. 7). Intriguingly, the mediation analysis highlighted that HCoV antibodies may have a direct effect on the prevention of symptoms in children. This is in agreement with previous data that observed an indirect link between symptoms and pre-existing HCoV antibody levels (28). Of note, as we solely analyzed HCoV S1 reactivity in our study, the influence of cross-reactivity observed must be considered as a lower limit. In particular, antibodies to S2, which is more conserved between HcoVs and SARS-CoV2 than S1, may further contribute to cross-reactivity (18).

Mucosal antibody responses, including specific early SARS-CoV-2 and cross-protective HCoV antibodies, co-operate to suppress early viral replication, underscoring the paramount importance of promoting mucosal immunity for defense (49, 83, 84). While natural HCoV immunity is clearly insufficient to prevent zoonotic coronavirus transmission, there may still be a cross-protective effect of HCoV immunity in limiting symptomatic SARS-CoV-2 disease as we show here. This finding provides encouragement for the development of pan-protective coronavirus vaccines.

## MATERIALS AND METHODS

### Human specimens

#### Longitudinal cohort study

The protocol and the epidemiological results of the longitudinal Ciao Corona cohort study (ClinicalTrials.gov identifier: NCT04448717) are reported elsewhere (56–59). Bi-monthly questionnaires with information on socio-demographics and flu-like symptoms (onset, type, duration) compatible with SARS-CoV-2 infection were available for most children. We considered children with at least one reported symptom to be symptomatic, while others were considered asymptomatic. Detailed information can be found in the supplemental material.

#### Definition of sub-cohorts from the longitudinal cohort

We defined sub-cohorts A, B, and C from the longitudinal Ciao Corona study as detailed in Fig. S2. Details are provided in the supplemental material.

#### Cross-sectional diagnostic cohort

The cohort comprises saliva samples from adults (*n* = 830) and children (*n* = 52) opting for a SARS-CoV-2 test at one of five participating test centers in the canton of Zurich, Switzerland, as part of a diagnostic survey study (64). Details are given in the supplemental material.

#### Saliva sample collection

For saliva collection, individuals of both cohorts were asked to clear their throat thoroughly and collect saliva into a supplied empty tube (64). Details are provided in the supplemental material.

### Reagents and cell lines

His-tagged SARS-CoV-2-derived antigens (RBD, S1, S2, N) and S1 of the four circulating HCoVs (HKU1, NL63, 229E, and OC43) were purchased from Sino Biological Europe GmbH, Eschborn, Germany (Table S4). Sources, specifics, and concentration of detection and control antibodies and sera used for the ABCORA and neutralization tests are listed in Table S5. HEK293-T cells were obtained from the American Type Culture Collection (ATCC CRL-11268) (107). HeLa ACE2 cell lines were purchased from Biogene, Shirley, NY. Both cell lines were cultured in Dulbecco's modified Eagle medium (DMEM) containing 10% fetal calf serum (FCS).

### Antibody measurements with multiplex bead assay ABCORA

Humoral response to SARS-CoV-2 was measured in EDTA plasma using the multiplex bead assay ABCORA as described (28). The assay measures IgG, IgA, and IgM reactivity to four SARS-CoV-2 antigens RBD, S1, S2, and N (12 SARS-CoV-2 parameters) in addition to IgG, IgA, and IgM reactivity to S1 of HCoV-HKU1. SARS-CoV-2-positive plasma reactivity using the ABCORA 2.3 computational approach achieves 98.20% specificity and 99.91% sensitivity (28).

### SARS-CoV-2 pseudo-neutralization assay

SARS-CoV-2 plasma neutralization activity against Wuhan-Hu-1 pseudoviruses was assessed using the HIV-1 reporter construct pHIV-1NL4-3 ΔEnv-NanoLuc (28) (pHIV-1Nanoluc, provided by P. Bieniasz, Rockefeller University, New York, NY, USA) and Human ACE2 Stable HeLa cell line (Biogene, Shirley, NY) and C-terminal truncated SARS-CoV-2 spike expression plasmids of strains P_CoV2_Wuhan, and Alpha, Delta, and Omicron BA.2. Pseudotyped viruses were produced in HEK293-T cells. Plasma and saliva neutralization titers causing 50% reduction in viral infectivity (NT50) in comparison with controls without plasma were calculated by fitting of a sigmoid dose-response curve (variable slope), using GraphPad Prism with constraints (bottom = 0, top = 100). If 50% inhibition was not achieved at the lowest plasma dilution of 1:100, a “less than” value was recorded. If 50% inhibition was not achieved at the lowest saliva dilution of 1:16, a “less than” value was recorded. All measurements were conducted in duplicate.

### Routine HCoV testing

Routine diagnostic analyses of respiratory samples for the endemic HCoVs (HKU1, NL63, 229E, and OC43) were performed in parallel with multiplex respiratory PCR panels (ePlex RP, Roche, or BioFire RP2.1, BioMérieux). Repetitive tests of the same patient within 20 days of the initial positive result were excluded.

### Statistical analysis

Analyses in the present study were designed and conducted retrospectively after the completion of the clinical studies. Summary of the different analyses that were conducted and the corresponding questions that we sought to answer are available in Table S1. All statistics obtained for these analyses are summarized in the supplementary material. Statistical analyses were performed in R (Version 4.0.5). Figures were made using the ggplot2 package (108). Details on the statistical analysis are provided in supplementary material (109–112).

## Data Availability

Data used to generate each figure are available in the supplemental material.

## SUPPLEMENTAL MATERIAL

Supplemental material mbio.02722-23-s0001.pdf Supplemental text, tables, and figures.

Source Data mbio.02722-23-s0002.xlsx Data used to generate each figure.

ASM does not own the copyrights to Supplemental Material that may be linked to, or accessed through, an article. The authors have granted ASM a non-exclusive, world-wide license to publish the Supplemental Material files. Please contact the corresponding author directly for reuse.

## References

[1] Wu Z, McGoogan JM. 2020. Characteristics of and important lessons from the coronavirus disease 2019 (COVID-19) outbreak in China: summary of a report of 72 314 cases from the Chinese center for disease control and prevention. JAMA 323:1239–1242. doi:10.1001/jama.2020.2648 32091533

[2] Buitrago-Garcia D, Egli-Gany D, Counotte MJ, Hossmann S, Imeri H, Ipekci AM, Salanti G, Low N. 2020. Occurrence and transmission potential of asymptomatic and presymptomatic SARS-CoV-2 infections: a living systematic review and meta-analysis. PLoS Med 17:e1003346. doi:10.1371/journal.pmed.1003346 32960881 PMC7508369

[3] Guan W-J, Ni Z-Y, Hu Y, Liang W-H, Ou C-Q, He J-X, Liu L, Shan H, Lei C-L, Hui DSC, et al.. 2020. Clinical characteristics of coronavirus disease 2019 in China. N Engl J Med 382:1708–1720. doi:10.1056/NEJMoa2002032 32109013 PMC7092819

[4] Argenziano MG, Bruce SL, Slater CL, Tiao JR, Baldwin MR, Barr RG, Chang BP, Chau KH, Choi JJ, Gavin N, et al.. 2020. Characterization and clinical course of 1000 patients with coronavirus disease 2019 in New York: retrospective case series. BMJ 369:m1996. doi:10.1136/bmj.m1996 32471884 PMC7256651

[5] Davis HE, McCorkell L, Vogel JM, Topol EJ. 2023. Long COVID: major findings, mechanisms and recommendations. Nat Rev Microbiol 21:133–146. doi:10.1038/s41579-023-00896-0 36639608 PMC9839201

[6] Crook H, Raza S, Nowell J, Young M, Edison P. 2021. Long covid-mechanisms, risk factors, and management. BMJ 374:n1648. doi:10.1136/bmj.n1648 34312178

[7] Merad M, Blish CA, Sallusto F, Iwasaki A. 2022. The immunology and immunopathology of COVID-19. Science 375:1122–1127. doi:10.1126/science.abm8108 35271343 PMC12828912

[8] Zhang Q, Bastard P, Liu Z, Le Pen J, Moncada-Velez M, Chen J, Ogishi M, Sabli IKD, Hodeib S, Korol C, et al.. 2020. Inborn errors of type I IFN immunity in patients with life-threatening COVID-19. Science 370:eabd4570. doi:10.1126/science.abd4570 32972995 PMC7857407

[9] Woodruff RC, Campbell AP, Taylor CA, Chai SJ, Kawasaki B, Meek J, Anderson EJ, Weigel A, Monroe ML, Reeg L, Bye E, Sosin DM, Muse A, Bennett NM, Billing LM, Sutton M, Talbot HK, McCaffrey K, Pham H, Patel K, Whitaker M, L McMorrow M, P Havers F. 2022. Risk factors for severe COVID-19 in children. Pediatrics 149:e2021053418. doi:10.1542/peds.2021-053418 34935038 PMC9213563

[10] Yek C, Warner S, Wiltz JL, Sun J, Adjei S, Mancera A, Silk BJ, Gundlapalli AV, Harris AM, Boehmer TK, Kadri SS. 2022. Risk factors for severe COVID-19 outcomes among persons aged ≥18 years who completed a primary COVID-19 vaccination series - 465 health care facilities, United States, December 2020-October 2021. MMWR Morb Mortal Wkly Rep 71:19–25. doi:10.15585/mmwr.mm7101a4 34990440 PMC8735560

[11] Subramanian A, Nirantharakumar K, Hughes S, Myles P, Williams T, Gokhale KM, Taverner T, Chandan JS, Brown K, Simms-Williams N, et al.. 2022. Symptoms and risk factors for long COVID in non-hospitalized adults. Nat Med 28:1706–1714. doi:10.1038/s41591-022-01909-w 35879616 PMC9388369

[12] Soares-Schanoski A, Sauerwald N, Goforth CW, Periasamy S, Weir DL, Lizewski S, Lizewski R, Ge Y, Kuzmina NA, Nair VD, Vangeti S, Marjanovic N, Cappuccio A, Cheng WS, Mofsowitz S, Miller CM, Yu XB, George M-C, Zaslavsky E, Bukreyev A, Troyanskaya OG, Sealfon SC, Letizia AG, Ramos I. 2022. Asymptomatic SARS-CoV-2 infection is associated with higher levels of serum IL-17C, matrix metalloproteinase 10 and fibroblast growth factors than mild symptomatic COVID-19. Front Immunol 13:821730. doi:10.3389/fimmu.2022.821730 35479098 PMC9037090

[13] Dobaño C, Alonso S, Fernández de Sevilla M, Vidal M, Jiménez A, Pons Tomas G, Jairoce C, Melé Casas M, Rubio R, Hernández García M, et al.. 2021. Antibody conversion rates to SARS-CoV-2 in saliva from children attending summer schools in Barcelona, Spain. BMC Med 19:309. doi:10.1186/s12916-021-02184-1 34809617 PMC8608564

[14] He J, Guo Y, Mao R, Zhang J. 2021. Proportion of asymptomatic coronavirus disease 2019: a systematic review and meta-analysis. J Med Virol 93:820–830. doi:10.1002/jmv.26326 32691881 PMC7404334

[15] North CM, Barczak A, Goldstein RH, Healy BC, Finkelstein DM, Ding DD, Kim A, Boucau J, Shaw B, Gilbert RF, et al.. 2022. Determining the incidence of asymptomatic SARS-CoV-2 among early recipients of COVID-19 vaccines (DISCOVER-COVID-19): a prospective cohort study of healthcare workers before, during and after vaccination. Clin Infect Dis 74:1275–1278. doi:10.1093/cid/ciab643 34363462 PMC8436402

[16] Dai L, Gao GF. 2021. Viral targets for vaccines against COVID-19. Nat Rev Immunol 21:73–82. doi:10.1038/s41577-020-00480-0 33340022 PMC7747004

[17] Giurgea LT, Han A, Memoli MJ. 2020. Universal coronavirus vaccines: the time to start is now. NPJ Vaccines 5:43. doi:10.1038/s41541-020-0198-1 32528732 PMC7256035

[18] Murray SM, Ansari AM, Frater J, Klenerman P, Dunachie S, Barnes E, Ogbe A. 2023. The impact of pre-existing cross-reactive immunity on SARS-CoV-2 infection and vaccine responses. Nat Rev Immunol 23:304–316. doi:10.1038/s41577-022-00809-x 36539527 PMC9765363

[19] Solomon MD, Escobar GJ, Lu Y, Schlessinger D, Steinman JB, Steinman L, Lee C, Liu VX. 2022. Risk of severe COVID-19 infection among adults with prior exposure to children. Proc Natl Acad Sci U S A 119:e2204141119. doi:10.1073/pnas.2204141119 35895714 PMC9388132

[20] Sayama Y, Okamoto M, Saito M, Saito-Obata M, Tamaki R, Joboco CD, Lupisan S, Oshitani H. 2023. Seroprevalence of four endemic human coronaviruses and, reactivity and neutralization capability against SARS-CoV-2 among children in the Philippines. Sci Rep 13:2310. doi:10.1038/s41598-023-29072-3 36759702 PMC9909632

[21] Loyal L, Braun J, Henze L, Kruse B, Dingeldey M, Reimer U, Kern F, Schwarz T, Mangold M, Unger C, et al.. 2021. Cross-reactive CD4^+^ T cells enhance SARS-CoV-2 immune responses upon infection and vaccination. Science 374:eabh1823. doi:10.1126/science.abh1823 34465633 PMC10026850

[22] Galipeau Y, Siragam V, Laroche G, Marion E, Greig M, McGuinty M, Booth RA, Durocher Y, Cuperlovic-Culf M, Bennett SAL, Crawley AM, Giguère PM, Cooper C, Langlois M-A. 2021. Relative ratios of human seasonal coronavirus antibodies predict the efficiency of cross-neutralization of SARS-CoV-2 spike binding to ACE2. EBioMedicine 74:103700. doi:10.1016/j.ebiom.2021.103700 34861490 PMC8629681

[23] Tso FY, Lidenge SJ, Peña PB, Clegg AA, Ngowi JR, Mwaiselage J, Ngalamika O, Julius P, West JT, Wood C. 2021. High prevalence of pre-existing serological cross-reactivity against severe acute respiratory syndrome coronavirus-2 (SARS-CoV-2) in sub-Saharan Africa. Int J Infect Dis 102:577–583. doi:10.1016/j.ijid.2020.10.104 33176202 PMC7648883

[24] Mateus J, Grifoni A, Tarke A, Sidney J, Ramirez SI, Dan JM, Burger ZC, Rawlings SA, Smith DM, Phillips E, et al.. 2020. Selective and cross-reactive SARS-CoV-2 T cell epitopes in unexposed humans. Science 370:89–94. doi:10.1126/science.abd3871 32753554 PMC7574914

[25] Dangi T, Palacio N, Sanchez S, Park M, Class J, Visvabharathy L, Ciucci T, Koralnik IJ, Richner JM, Penaloza-MacMaster P. 2021. Cross-protective immunity following coronavirus vaccination and coronavirus infection. J Clin Invest 131:e151969. doi:10.1172/JCI151969 34623973 PMC8670840

[26] Stoddard CI, Galloway J, Chu HY, Shipley MM, Sung K, Itell HL, Wolf CR, Logue JK, Magedson A, Garrett ME, Crawford KHD, Laserson U, Matsen FA, Overbaugh J. 2021. Epitope profiling reveals binding signatures of SARS-CoV-2 immune response in natural infection and cross-reactivity with endemic human CoVs. Cell Rep 35:109164. doi:10.1016/j.celrep.2021.109164 33991511 PMC8121454

[27] Ortega N, Ribes M, Vidal M, Rubio R, Aguilar R, Williams S, Barrios D, Alonso S, Hernández-Luis P, Mitchell RA, et al.. 2021. Seven-month kinetics of SARS-CoV-2 antibodies and role of pre-existing antibodies to human coronaviruses. Nat Commun 12:4740. doi:10.1038/s41467-021-24979-9 34362897 PMC8346582

[28] Abela IA, Pasin C, Schwarzmüller M, Epp S, Sickmann ME, Schanz MM, Rusert P, Weber J, Schmutz S, Audigé A, et al.. 2021. Multifactorial seroprofiling dissects the contribution of pre-existing human coronaviruses responses to SARS-CoV-2 immunity. Nat Commun 12:6703. doi:10.1038/s41467-021-27040-x 34795285 PMC8602384

[29] Brown JA, Hauser A, Abela IA, Pasin C, Epp S, Mohloanyane T, Nsakala BL, Trkola A, Labhardt ND, Kouyos RD, Günthard HF. 2023. Seroprofiling of antibodies against endemic human coronaviruses and severe acute respiratory syndrome coronavirus 2 in a human immunodeficiency virus cohort in Lesotho: correlates of antibody response and seropositivity. J Infect Dis 228:1042–1054. doi:10.1093/infdis/jiad197 37261930 PMC10582919

[30] Brodin P. 2022. SARS-CoV-2 infections in children: understanding diverse outcomes. Immunity 55:201–209. doi:10.1016/j.immuni.2022.01.014 35093190 PMC8769938

[31] Khan T, Rahman M, Ali FA, Huang SSY, Ata M, Zhang Q, Bastard P, Liu Z, Jouanguy E, Béziat V, Cobat A, Nasrallah GK, Yassine HM, Smatti MK, Saeed A, Vandernoot I, Goffard J-C, Smits G, Migeotte I, Haerynck F, Meyts I, Abel L, Casanova J-L, Hasan MR, Marr N. 2021. Distinct antibody repertoires against endemic human coronaviruses in children and adults. JCI Insight 6:e144499. doi:10.1172/jci.insight.144499 33497357 PMC7934927

[32] Nogrady B. 2020. How kids' immune systems can evade COVID. Nature 588:382. doi:10.1038/d41586-020-03496-7 33303982

[33] Wratil PR, Schmacke NA, Karakoc B, Dulovic A, Junker D, Becker M, Rothbauer U, Osterman A, Spaeth PM, Ruhle A, et al.. 2021. Evidence for increased SARS-CoV-2 susceptibility and COVID-19 severity related to pre-existing immunity to seasonal coronaviruses. Cell Rep 37:110169. doi:10.1016/j.celrep.2021.110169 34932974 PMC8648802

[34] Anderson EM, Goodwin EC, Verma A, Arevalo CP, Bolton MJ, Weirick ME, Gouma S, McAllister CM, Christensen SR, Weaver J, et al.. 2021. Seasonal human coronavirus antibodies are boosted upon SARS-CoV-2 infection but not associated with protection. Cell 184:1858–1864. doi:10.1016/j.cell.2021.02.010 33631096 PMC7871851

[35] Park S, Lee Y, Michelow IC, Choe YJ. 2020. Global seasonality of human coronaviruses: a systematic review. Open Forum Infect Dis 7:ofaa443. doi:10.1093/ofid/ofaa443 33204751 PMC7651300

[36] Shah MM, Winn A, Dahl RM, Kniss KL, Silk BJ, Killerby ME. 2022. Seasonality of common human coronaviruses, United States, 2014-2021^1^. Emerg Infect Dis 28:1970–1976. doi:10.3201/eid2810.220396 36007923 PMC9514339

[37] Friedman N, Alter H, Hindiyeh M, Mendelson E, Shemer Avni Y, Mandelboim M. 2018. Human coronavirus infections in Israel: epidemiology, clinical symptoms and summer seasonality of HCoV-HKU1. Viruses 10:515. doi:10.3390/v10100515 30241410 PMC6213580

[38] Earle KA, Ambrosino DM, Fiore-Gartland A, Goldblatt D, Gilbert PB, Siber GR, Dull P, Plotkin SA. 2021. Evidence for antibody as a protective correlate for COVID-19 vaccines. Vaccine 39:4423–4428. doi:10.1016/j.vaccine.2021.05.063 34210573 PMC8142841

[39] Röltgen K, Boyd SD. 2021. Antibody and B cell responses to SARS-CoV-2 infection and vaccination. Cell Host Microbe 29:1063–1075. doi:10.1016/j.chom.2021.06.009 34174992 PMC8233571

[40] Khoury DS, Cromer D, Reynaldi A, Schlub TE, Wheatley AK, Juno JA, Subbarao K, Kent SJ, Triccas JA, Davenport MP. 2021. Neutralizing antibody levels are highly predictive of immune protection from symptomatic SARS-CoV-2 infection. Nat Med 27:1205–1211. doi:10.1038/s41591-021-01377-8 34002089

[41] Hall VJ, Foulkes S, Charlett A, Atti A, Monk EJM, Simmons R, Wellington E, Cole MJ, Saei A, Oguti B, Munro K, Wallace S, Kirwan PD, Shrotri M, Vusirikala A, Rokadiya S, Kall M, Zambon M, Ramsay M, Brooks T, Brown CS, Chand MA, Hopkins S, SIREN Study Group. 2021. SARS-CoV-2 infection rates of antibody-positive compared with antibody-negative health-care workers in England: a large, multicentre, prospective cohort study (SIREN). Lancet 397:1459–1469. doi:10.1016/S0140-6736(21)00675-9 33844963 PMC8040523

[42] Zou L, Ruan F, Huang M, Liang L, Huang H, Hong Z, Yu J, Kang M, Song Y, Xia J, Guo Q, Song T, He J, Yen H-L, Peiris M, Wu J. 2020. SARS-CoV-2 viral load in upper respiratory specimens of infected patients. N Engl J Med 382:1177–1179. doi:10.1056/NEJMc2001737 32074444 PMC7121626

[43] Huang N, Pérez P, Kato T, Mikami Y, Okuda K, Gilmore RC, Conde CD, Gasmi B, Stein S, Beach M, et al.. 2021. SARS-CoV-2 infection of the oral cavity and saliva. Nat Med 27:892–903. doi:10.1038/s41591-021-01296-8 33767405 PMC8240394

[44] Isho B, Abe KT, Zuo M, Jamal AJ, Rathod B, Wang JH, Li Z, Chao G, Rojas OL, Bang YM, et al.. 2020. Persistence of serum and saliva antibody responses to SARS-CoV-2 spike antigens in COVID-19 patients. Sci Immunol 5:52. doi:10.1126/sciimmunol.abe5511 PMC8050884 33033173

[45] Russell MW, Moldoveanu Z, Ogra PL, Mestecky J. 2020. Mucosal immunity in COVID-19: a neglected but critical aspect of SARS-CoV-2 infection. Front Immunol 11:611337. doi:10.3389/fimmu.2020.611337 33329607 PMC7733922

[46] Sette A, Crotty S. 2021. Adaptive immunity to SARS-CoV-2 and COVID-19. Cell 184:861–880. doi:10.1016/j.cell.2021.01.007 33497610 PMC7803150

[47] Pisanic N, Randad PR, Kruczynski K, Manabe YC, Thomas DL, Pekosz A, Klein SL, Betenbaugh MJ, Clarke WA, Laeyendecker O, Caturegli PP, Larman HB, Detrick B, Fairley JK, Sherman AC, Rouphael N, Edupuganti S, Granger DA, Granger SW, Collins MH, Heaney CD. 2020. COVID-19 serology at population scale: SARS-CoV-2-specific antibody responses in saliva. J Clin Microbiol 59:e02204-20. doi:10.1128/JCM.02204-20 33067270 PMC7771435

[48] Pinilla YT, Heinzel C, Caminada L-F, Consolaro D, Esen M, Kremsner PG, Held J, Kreidenweiss A, Fendel R. 2021. SARS-CoV-2 antibodies are persisting in saliva for more than 15 months after infection and become strongly boosted after vaccination. Front Immunol 12:798859. doi:10.3389/fimmu.2021.798859 34956236 PMC8695841

[49] Sterlin D, Mathian A, Miyara M, Mohr A, Anna F, Claër L, Quentric P, Fadlallah J, Devilliers H, Ghillani P, et al.. 2021. IgA dominates the early neutralizing antibody response to SARS-CoV-2. Sci Transl Med 13:eabd2223. doi:10.1126/scitranslmed.abd2223 33288662 PMC7857408

[50] Becker M, Dulovic A, Junker D, Ruetalo N, Kaiser PD, Pinilla YT, Heinzel C, Haering J, Traenkle B, Wagner TR, et al.. 2021. Immune response to SARS-CoV-2 variants of concern in vaccinated individuals. Nat Commun 12:3109. doi:10.1038/s41467-021-23473-6 34035301 PMC8149389

[51] Ketas TJ, Chaturbhuj D, Portillo VMC, Francomano E, Golden E, Chandrasekhar S, Debnath G, Díaz-Tapia R, Yasmeen A, Kramer KD, Munawar T, Leconet W, Zhao Z, Brouwer PJM, Cushing MM, Sanders RW, Cupo A, Klasse PJ, Formenti SC, Moore JP. 2021. Antibody responses to SARS-CoV-2 mRNA vaccines are detectable in saliva. Pathog Immun 6:116–134. doi:10.20411/pai.v6i1.441 34136730 PMC8201795

[52] Azzi L, Dalla Gasperina D, Veronesi G, Shallak M, Ietto G, Iovino D, Baj A, Gianfagna F, Maurino V, Focosi D, Maggi F, Ferrario MM, Dentali F, Carcano G, Tagliabue A, Maffioli LS, Accolla RS, Forlani G. 2022. Mucosal immune response in BNT162b2 COVID-19 vaccine recipients. EBioMedicine 75:103788. doi:10.1016/j.ebiom.2021.103788 34954658 PMC8718969

[53] Sokal A, Chappert P, Barba-Spaeth G, Roeser A, Fourati S, Azzaoui I, Vandenberghe A, Fernandez I, Meola A, Bouvier-Alias M, et al.. 2021. Maturation and persistence of the anti-SARS-CoV-2 memory B cell response. Cell 184:1201–1213. doi:10.1016/j.cell.2021.01.050 33571429 PMC7994111

[54] Ng KW, Faulkner N, Cornish GH, Rosa A, Harvey R, Hussain S, Ulferts R, Earl C, Wrobel AG, Benton DJ, et al.. 2020. Preexisting and de novo humoral immunity to SARS-CoV-2 in humans. Science 370:1339–1343. doi:10.1126/science.abe1107 33159009 PMC7857411

[55] Marzi R, Bassi J, Silacci-Fregni C, Bartha I, Muoio F, Culap K, Sprugasci N, Lombardo G, Saliba C, Cameroni E, et al.. 2023. Maturation of SARS-CoV-2 spike-specific memory B cells drives resilience to viral escape. iScience 26:105726. doi:10.1016/j.isci.2022.105726 36507220 PMC9721160

[56] Ulyte A, Radtke T, Abela IA, Haile SR, Ammann P, Berger C, Trkola A, Fehr J, Puhan MA, Kriemler S. 2021. Evolution of SARS-CoV-2 seroprevalence and clusters in school children from June 2020 to April 2021: prospective cohort study Ciao Corona. Swiss Med Wkly 151:w30092. doi:10.4414/smw.2021.w30092 34797618

[57] Ulyte A, Radtke T, Abela IA, Haile SR, Berger C, Huber M, Schanz M, Schwarzmueller M, Trkola A, Fehr J, Puhan MA, Kriemler S. 2021. Clustering and longitudinal change in SARS-CoV-2 seroprevalence in school children in the canton of Zurich, Switzerland: prospective cohort study of 55 schools. BMJ 372:n616. doi:10.1136/bmj.n616 33731327 PMC7966948

[58] Ulyte A, Radtke T, Abela IA, Haile SR, Blankenberger J, Jung R, Capelli C, Berger C, Frei A, Huber M, Schanz M, Schwarzmueller M, Trkola A, Fehr J, Puhan MA, Kriemler S. 2021. Variation in SARS-CoV-2 seroprevalence across districts, schools and classes: baseline measurements from a cohort of primary and secondary school children in Switzerland. BMJ Open 11:e047483. doi:10.1136/bmjopen-2020-047483 PMC8316698 34312201

[59] Ulyte A, Radtke T, Abela IA, Haile SR, Braun J, Jung R, Berger C, Trkola A, Fehr J, Puhan MA, Kriemler S. 2020. Seroprevalence and immunity of SARS-CoV-2 infection in children and adolescents in schools in Switzerland: design for a longitudinal, school-based prospective cohort study. Int J Public Health 65:1549–1557. doi:10.1007/s00038-020-01495-z 33063141 PMC7561232

[60] Speich B, Chammartin F, Abela IA, Amico P, Stoeckle MP, Eichenberger AL, Hasse B, Braun DL, Schuurmans MM, Müller TF, Tamm M, Audigé A, Mueller NJ, Rauch A, Günthard HF, Koller MT, Trkola A, Briel M, Kusejko K, Bucher HC, Swiss HIV Cohort Study and the Swiss Transplant Cohort Study. 2022. Antibody response in immunocompromised patients after the administration of SARS-CoV-2 vaccine BNT162b2 or mRNA-1273: a randomised controlled trial. Clin Infect Dis 75:e585–e593. doi:10.1093/cid/ciac169 35234868 PMC8903480

[61] Marconato M, Abela IA, Hauser A, Schwarzmüller M, Katzensteiner R, Braun DL, Epp S, Audigé A, Weber J, Rusert P, Schindler E, Pasin C, West E, Böni J, Kufner V, Huber M, Zaheri M, Schmutz S, Frey BM, Kouyos RD, Günthard HF, Manz MG, Trkola A. 2022. Antibodies from convalescent plasma promote SARS-CoV-2 clearance in individuals with and without endogenous antibody response. J Clin Invest 132:12. doi:10.1172/JCI158190 PMC9197521 35482408

[62] Chammartin F, Kusejko K, Pasin C, Trkola A, Briel M, Amico P, Stoekle MP, Eichenberger AL, Hasse B, Braun DL, Schuurmans MM, Müller TF, Tamm M, Mueller NJ, Rauch A, Koller MT, Günthard HF, Bucher HC, Speich B, Abela IA, and the Swiss HIV Cohort Study. 2022. Determinants of antibody response to severe acute respiratory syndrome coronavirus 2 mRNA vaccines in people with HIV. AIDS 36:1465–1468. doi:10.1097/QAD.0000000000003246 35876706

[63] Huang A, Cicin-Sain C, Pasin C, Epp S, Audigé A, Müller NJ, Nilsson J, Bankova A, Wolfensberger N, Vilinovszki O, Nair G, Hockl P, Schanz U, Kouyos RD, Hasse B, Zinkernagel AS, Trkola A, Manz MG, Abela IA, Müller AMS. 2022. Antibody response to SARS-CoV-2 vaccination in patients following allogeneic hematopoietic cell transplantation. Transplant Cell Ther 28:214. doi:10.1016/j.jtct.2022.01.019 PMC8802693 35092892

[64] Huber M, Schreiber PW, Scheier T, Audigé A, Buonomano R, Rudiger A, Braun DL, Eich G, Keller DI, Hasse B, Böni J, Berger C, Günthard HF, Manrique A, Trkola A. 2021. High efficacy of saliva in detecting SARS-CoV-2 by RT-PCR in adults and children. Microorganisms 9:642. doi:10.3390/microorganisms9030642 33808815 PMC8003663

[65] Bundesamt für Gesundheit BAG. 2022. Covid-⁠19 schweiz. Available from: https://www.covid19.admin.ch/de/epidemiologic/case?geo=ZH

[66] Fenwick C, Croxatto A, Coste AT, Pojer F, André C, Pellaton C, Farina A, Campos J, Hacker D, Lau K, Bosch B-J, Gonseth Nussle S, Bochud M, D’Acremont V, Trono D, Greub G, Pantaleo G. 2021. Changes in SARS-CoV-2 spike versus nucleoprotein antibody responses impact the estimates of infections in population-based seroprevalence studies. J Virol 95:e01828-20. doi:10.1128/JVI.01828-20 33144321 PMC7925109

[67] Sette A, Crotty S. 2020. Pre-existing immunity to SARS-CoV-2: the knowns and unknowns. Nat Rev Immunol 20:457–458. doi:10.1038/s41577-020-0389-z 32636479 PMC7339790

[68] Gould VMW, Francis JN, Anderson KJ, Georges B, Cope AV, Tregoning JS. 2017. Nasal IgA provides protection against human influenza challenge in volunteers with low serum influenza antibody titre. Front Microbiol 8:900. doi:10.3389/fmicb.2017.00900 28567036 PMC5434144

[69] Ogra PL. 2004. Respiratory syncytial virus: the virus, the disease and the immune response. Paediatr Respir Rev 5:S119–S126. doi:10.1016/s1526-0542(04)90023-1 14980256 PMC7172639

[70] Weinberg A, Song L-Y, Walker R, Allende M, Fenton T, Patterson-Bartlett J, Nachman S, Kemble G, Yi T-T, Defechereux P, Wara D, Read JS, Levin M, IMPAACT P1057 Team. 2010. Anti-influenza serum and mucosal antibody responses after administration of live attenuated or inactivated influenza vaccines to HIV-infected children. J Acquir Immune Defic Syndr 55:189–196. doi:10.1097/QAI.0b013e3181e46308 20581690 PMC3290334

[71] Muecksch F, Weisblum Y, Barnes CO, Schmidt F, Schaefer-Babajew D, Wang Z, C Lorenzi JC, Flyak AI, DeLaitsch AT, Huey-Tubman KE, et al.. 2021. Affinity maturation of SARS-CoV-2 neutralizing antibodies confers potency, breadth, and resilience to viral escape mutations. Immunity 54:1853–1868. doi:10.1016/j.immuni.2021.07.008 34331873 PMC8323339

[72] Sakharkar M, Rappazzo CG, Wieland-Alter WF, Hsieh C-L, Wrapp D, Esterman ES, Kaku CI, Wec AZ, Geoghegan JC, McLellan JS, Connor RI, Wright PF, Walker LM. 2021. Prolonged evolution of the human B cell response to SARS-CoV-2 infection. Sci Immunol 6:eabg6916. doi:10.1126/sciimmunol.abg6916 33622975 PMC8128290

[73] Planchais C, Fernández I, Bruel T, de Melo GD, Prot M, Beretta M, Guardado-Calvo P, Dufloo J, Molinos-Albert LM, Backovic M, et al.. 2022. Potent human broadly SARS-CoV-2-neutralizing IgA and IgG antibodies effective against Omicron BA.1 and BA.2. J Exp Med 219:e20220638. doi:10.1084/jem.20220638 35704748 PMC9206116

[74] Dufloo J, Grzelak L, Staropoli I, Madec Y, Tondeur L, Anna F, Pelleau S, Wiedemann A, Planchais C, Buchrieser J, Robinot R, Ungeheuer M-N, Mouquet H, Charneau P, White M, Lévy Y, Hoen B, Fontanet A, Schwartz O, Bruel T. 2021. Asymptomatic and symptomatic SARS-CoV-2 infections elicit polyfunctional antibodies. Cell Rep Med 2:100275. doi:10.1016/j.xcrm.2021.100275 33899033 PMC8057765

[75] Ng DL, Goldgof GM, Shy BR, Levine AG, Balcerek J, Bapat SP, Prostko J, Rodgers M, Coller K, Pearce S, et al.. 2020. SARS-CoV-2 seroprevalence and neutralizing activity in donor and patient blood. Nat Commun 11:4698. doi:10.1038/s41467-020-18468-8 32943630 PMC7499171

[76] Yang Y, Yang M, Peng Y, Liang Y, Wei J, Xing L, Guo L, Li X, Li J, Wang J, Li M, Xu Z, Zhang M, Wang F, Shi Y, Yuan J, Liu Y. 2022. Longitudinal analysis of antibody dynamics in COVID-19 convalescents reveals neutralizing responses up to 16 months after infection. Nat Microbiol 7:423–433. doi:10.1038/s41564-021-01051-2 35132197

[77] Bekliz M, Adea K, Vetter P, Eberhardt CS, Hosszu-Fellous K, Vu D-L, Puhach O, Essaidi-Laziosi M, Waldvogel-Abramowski S, Stephan C, L’Huillier AG, Siegrist C-A, Didierlaurent AM, Kaiser L, Meyer B, Eckerle I. 2022. Neutralization capacity of antibodies elicited through homologous or heterologous infection or vaccination against SARS-CoV-2 VOCs. Nat Commun 13:3840. doi:10.1038/s41467-022-31556-1 35787633 PMC9253337

[78] Dupont L, Snell LB, Graham C, Seow J, Merrick B, Lechmere T, Maguire TJA, Hallett SR, Pickering S, Charalampous T, et al.. 2021. Neutralizing antibody activity in convalescent sera from infection in humans with SARS-CoV-2 and variants of concern. Nat Microbiol 6:1433–1442. doi:10.1038/s41564-021-00974-0 34654917 PMC8556155

[79] Newman J, Thakur N, Peacock TP, Bialy D, Elrefaey AME, Bogaardt C, Horton DL, Ho S, Kankeyan T, Carr C, Hoschler K, Barclay WS, Amirthalingam G, Brown KE, Charleston B, Bailey D. 2022. Neutralizing antibody activity against 21 SARS-CoV-2 variants in older adults vaccinated with BNT162b2. Nat Microbiol 7:1180–1188. doi:10.1038/s41564-022-01163-3 35836002 PMC9352594

[80] Bean DJ, Sagar M. 2021. Family matters for coronavirus disease and vaccines. J Clin Invest 131:e155615. doi:10.1172/JCI155615 34752421 PMC8670830

[81] Ye Z-W, Yuan S, Yuen K-S, Fung S-Y, Chan C-P, Jin D-Y. 2020. Zoonotic origins of human coronaviruses. Int J Biol Sci 16:1686–1697. doi:10.7150/ijbs.45472 32226286 PMC7098031

[82] Focosi D, Maggi F, Casadevall A. 2022. Mucosal vaccines, sterilizing immunity, and the future of SARS-CoV-2 virulence. Viruses 14:187. doi:10.3390/v14020187 35215783 PMC8878800

[83] Mettelman RC, Allen EK, Thomas PG. 2022. Mucosal immune responses to infection and vaccination in the respiratory tract. Immunity 55:749–780. doi:10.1016/j.immuni.2022.04.013 35545027 PMC9087965

[84] Sheikh-Mohamed S, Sanders EC, Gommerman JL, Tal MC. 2022. Guardians of the oral and nasopharyngeal galaxy: IgA and protection against SARS-CoV-2 infection. Immunol Rev 309:75–85. doi:10.1111/imr.13118 35815463 PMC9349649

[85] Nguyen-Contant P, Embong AK, Kanagaiah P, Chaves FA, Yang H, Branche AR, Topham DJ, Sangster MY. 2020. S protein-reactive IgG and memory B cell production after human SARS-CoV-2 infection includes broad reactivity to the S2 subunit. mBio 11:e01991-20. doi:10.1128/mBio.01991-20 32978311 PMC7520599

[86] Zhou P, Song G, Liu H, Yuan M, He W-T, Beutler N, Zhu X, Tse LV, Martinez DR, Schäfer A, et al.. 2023. Broadly neutralizing anti-S2 antibodies protect against all three human betacoronaviruses that cause deadly disease. Immunity 56:669–686. doi:10.1016/j.immuni.2023.02.005 36889306 PMC9933850

[87] Ko S-H, Chen W-Y, Su S-C, Lin H-T, Ke F-Y, Liang K-H, Hsu F-F, Kumari M, Fu C-Y, Wu H-C. 2022. Monoclonal antibodies against S2 subunit of spike protein exhibit broad reactivity toward SARS-CoV-2 variants. J Biomed Sci 29:108. doi:10.1186/s12929-022-00891-2 36550570 PMC9774083

[88] Braun J, Loyal L, Frentsch M, Wendisch D, Georg P, Kurth F, Hippenstiel S, Dingeldey M, Kruse B, Fauchere F, et al.. 2020. SARS-CoV-2-reactive T cells in healthy donors and patients with COVID-19. Nature 587:270–274. doi:10.1038/s41586-020-2598-9 32726801

[89] Israel A, Shenhar Y, Green I, Merzon E, Golan-Cohen A, Schäffer AA, Ruppin E, Vinker S, Magen E. 2021. Large-scale study of antibody titer decay following BNT162b2 mRNA vaccine or SARS-CoV-2 infection. Vaccines (Basel) 10:64. doi:10.3390/vaccines10010064 35062724 PMC8781423

[90] Naaber P, Tserel L, Kangro K, Sepp E, Jürjenson V, Adamson A, Haljasmägi L, Rumm AP, Maruste R, Kärner J, Gerhold JM, Planken A, Ustav M, Kisand K, Peterson P. 2021. Dynamics of antibody response to BNT162b2 vaccine after six months: a longitudinal prospective study. Lancet Reg Health Eur 10:100208. doi:10.1016/j.lanepe.2021.100208 34514454 PMC8418937

[91] Lyke KE, Atmar RL, Islas CD, Posavad CM, Szydlo D, Paul Chourdhury R, Deming ME, Eaton A, Jackson LA, Branche AR, et al.. 2022. Rapid decline in vaccine-boosted neutralizing antibodies against SARS-CoV-2 Omicron variant. Cell Rep Med 3:100679. doi:10.1016/j.xcrm.2022.100679 35798000 PMC9212999

[92] Havervall S, Marking U, Svensson J, Greilert-Norin N, Bacchus P, Nilsson P, Hober S, Gordon M, Blom K, Klingström J, Åberg M, Smed-Sörensen A, Thålin C. 2022. Anti-spike mucosal IgA protection against SARS-CoV-2 Omicron infection. N Engl J Med 387:1333–1336. doi:10.1056/NEJMc2209651 36103621 PMC9511632

[93] Mouro V, Fischer A. 2022. Dealing with a mucosal viral pandemic: lessons from COVID-19 vaccines. Mucosal Immunol 15:584–594. doi:10.1038/s41385-022-00517-8 35505121 PMC9062288

[94] Lund FE, Randall TD. 2021. Scent of a vaccine. Science 373:397–399. doi:10.1126/science.abg9857 34437109

[95] Lapuente D, Fuchs J, Willar J, Vieira Antão A, Eberlein V, Uhlig N, Issmail L, Schmidt A, Oltmanns F, Peter AS, Mueller-Schmucker S, Irrgang P, Fraedrich K, Cara A, Hoffmann M, Pöhlmann S, Ensser A, Pertl C, Willert T, Thirion C, Grunwald T, Überla K, Tenbusch M. 2021. Protective mucosal immunity against SARS-CoV-2 after heterologous systemic prime-mucosal boost immunization. Nat Commun 12:6871. doi:10.1038/s41467-021-27063-4 34836955 PMC8626513

[96] Mao T, Israelow B, Peña-Hernández MA, Suberi A, Zhou L, Luyten S, Reschke M, Dong H, Homer RJ, Saltzman WM, Iwasaki A. 2022. Unadjuvanted intranasal spike vaccine elicits protective mucosal immunity against sarbecoviruses. Science 378:eabo2523. doi:10.1126/science.abo2523 36302057 PMC9798903

[97] Ku Z, Xie X, Hinton PR, Liu X, Ye X, Muruato AE, Ng DC, Biswas S, Zou J, Liu Y, Pandya D, Menachery VD, Rahman S, Cao Y-A, Deng H, Xiong W, Carlin KB, Liu J, Su H, Haanes EJ, Keyt BA, Zhang N, Carroll SF, Shi P-Y, An Z. 2021. Nasal delivery of an IgM offers broad protection from SARS-CoV-2 variants. Nature 595:718–723. doi:10.1038/s41586-021-03673-2 34082438 PMC8742224

[98] Sagar M, Reifler K, Rossi M, Miller NS, Sinha P, White LF, Mizgerd JP. 2021. Recent endemic coronavirus infection is associated with less-severe COVID-19. J Clin Invest 131:e143380. doi:10.1172/JCI143380 32997649 PMC7773342

[99] Waterlow NR, van Leeuwen E, Davies NG, Flasche S, Eggo RM, CMMID COVID-19 Working Group. 2021. How immunity from and interaction with seasonal coronaviruses can shape SARS-CoV-2 epidemiology. Proc Natl Acad Sci U S A 118:49. doi:10.1073/pnas.2108395118 PMC8670441 34873059

[100] Aran D, Beachler DC, Lanes S, Overhage JM. 2020. Prior presumed coronavirus infection reduces COVID-19 risk: a cohort study. J Infect 81:923–930. doi:10.1016/j.jinf.2020.10.023 33127456 PMC7590640

[101] Callow KA, Parry HF, Sergeant M, Tyrrell DA. 1990. The time course of the immune response to experimental coronavirus infection of man. Epidemiol Infect 105:435–446. doi:10.1017/s0950268800048019 2170159 PMC2271881

[102] Petrie JG, Bazzi LA, McDermott AB, Follmann D, Esposito D, Hatcher C, Mateja A, Narpala SR, O’Connell SE, Martin ET, Monto AS. 2021. Coronavirus occurrence in the household influenza vaccine evaluation (HIVE) cohort of Michigan households: reinfection frequency and serologic responses to seasonal and severe acute respiratory syndrome coronaviruses. J Infect Dis 224:49–59. doi:10.1093/infdis/jiab161 33755731 PMC8083771

[103] Edridge AWD, Kaczorowska J, Hoste ACR, Bakker M, Klein M, Loens K, Jebbink MF, Matser A, Kinsella CM, Rueda P, Ieven M, Goossens H, Prins M, Sastre P, Deijs M, van der Hoek L. 2020. Seasonal coronavirus protective immunity is short-lasting. Nat Med 26:1691–1693. doi:10.1038/s41591-020-1083-1 32929268

[104] Rees EM, Waterlow NR, Lowe R, Kucharski AJ, Centre for the Mathematical Modelling of Infectious Diseases COVID-19 Working Group. 2021. Estimating the duration of seropositivity of human seasonal coronaviruses using seroprevalence studies. Wellcome Open Res 6:138. doi:10.12688/wellcomeopenres.16701.3 34708157 PMC8517721

[105] Hamady A, Lee J, Loboda ZA. 2022. Waning antibody responses in COVID-19: what can we learn from the analysis of other coronaviruses? Infection 50:11–25. doi:10.1007/s15010-021-01664-z 34324165 PMC8319587

[106] Dowell AC, Butler MS, Jinks E, Tut G, Lancaster T, Sylla P, Begum J, Bruton R, Pearce H, Verma K, et al.. 2022. Children develop robust and sustained cross-reactive spike-specific immune responses to SARS-CoV-2 infection. Nat Immunol 23:40–49. doi:10.1038/s41590-021-01089-8 34937928 PMC8709786

[107] Wei X, Decker JM, Liu H, Zhang Z, Arani RB, Kilby JM, Saag MS, Wu X, Shaw GM, Kappes JC. 2002. Emergence of resistant human immunodeficiency virus type 1 in patients receiving fusion inhibitor (T-20) monotherapy. Antimicrob Agents Chemother 46:1896–1905. doi:10.1128/AAC.46.6.1896-1905.2002 12019106 PMC127242

[108] Wickham H. 2016. ggplot2: elegant graphics for data analysis. Cham.

[109] Rothman KJ. 1990. No adjustments are needed for multiple comparisons. Epidemiology 1:43–46. doi:10.1097/00001648-199001000-00010 2081237

[110] Henningsen A. 2022. Censored regression (tobit) models. Available from: http://www.sampleselection.org

[111] Tingley D, Yamamoto T, Hirose K, Keele L, Imai K. 2014. Mediation: R package for causal mediation analysis. J Stat Softw 59:1–38. doi:10.18637/jss.v059.i0526917999

[112] Li B, Qingzhao Y. 2022. mma: multiple mediation analysis. https://www.r-project.org.

