# Supplementary material for: Cross-protective HCoV immunity reduces symptom development during SARS-CoV-2 infection
Source: mBio. 2024 Jan 25;15(2):e02722-23. doi: 10.1128/mbio.02722-23 (PMC10865973; doi:10.1128/mbio.02722-23)
Supplement: Supplemental material — Supplemental text, tables, and figures. [file mbio.02722-23-s0001.pdf]

Supplementary Material

Content

Supplementary Material 1
1. Supplementary text for material and methods 1
   1. Longitudinal cohort study 1
   2. Definition of sub-cohorts from the longitudinal cohort 2
   3. Cross-sectional diagnostic cohort 2
   4. Saliva sample collection 3
   5. Statistical analysis 3
2. Supplementary Tables 5
   Supplementary Table 1. Analysis summary. 5
   Supplementary Table 2. Description of the longitudinal cohort. Samples from children were repeatedly collected during three testing rounds (R1-R3). 6
   Supplementary Table 3. Description of the cross-sectional diagnostic cohort. 7
   Supplementary Table 4. CoV-derived antigens. 8
   Supplementary Table 5. Origin and characteristics of antibody reagents. 9
3. Supplementary Figures 9

1. Supplementary text for material and methods

1. Longitudinal cohort study

The study was based in the canton of Zurich, Switzerland. 55 primary schools, stratified by region participated and classes within participating schools were randomly selected. Venous blood samples were repeatedly collected from participants at schools during three testing rounds. Study entry was allowed at all collection time points, thus not for all participants a full longitudinal sample and data collection occurred. Saliva was collected in parallel to blood for the first two collection rounds. The following previously established plasma antibody data [56-59] of the three testing rounds were available for analysis in the present study: from the 1st collection round (16 June to 9 July 2020) we analyzed 2505 participants with plasma serology data, from the 2nd testing round (26 October to 19 November 2020) 2526 participants, and from the 3rd round (15 March to 16 April 2021)

2455 participants. Saliva samples collected during the first two rounds that were available for antibody measurements in the present study included 2489 samples from the 1st collection round and 2455 samples from the 2nd round. Bi-monthly questionnaires with information on socio-demographics and flu-like symptoms (onset, type, duration) compatible with SARS-CoV-2 infection were available for most children [56-59]. We considered children with at least one reported symptom to be symptomatic, while others are considered asymptomatic. To assess the impact of HCoV antibodies on symptoms, we only considered the follow up questionnaires collected after the last SARS-CoV-2 negative and before the first SARS-CoV-2 positive serological measurement.

2. Definition of sub-cohorts from the longitudinal cohort

We defined sub-cohorts A, B and C from the longitudinal Ciao corona study as detailed in Supplementary Figure 2. Details are provided in Supplementary Material. Since not all participants participated at all sampling round, longitudinal comparisons were restricted to those where consecutive plasma or saliva collections were available. Sub-cohort A was constituted of children who were SARS-CoV-2 seropositive at the round 1 of testing (June-July 2020). Sub-cohort B was constituted of children who were SARS-CoV-2 seronegative at the round 1 of testing and SARS-CoV-2 seropositive at the round 2 of testing (October-November 2020). Sub-cohort C was constituted of children who were SARS-CoV-2 seronegative at both rounds 1 and 2, and SARS-CoV-2 seropositive for round 3 (March-April 2023).

3. Cross-sectional diagnostic cohort

The cohort comprises saliva samples from adults (n = 830) and children (n = 52) opting for a SARS-CoV-2 test at one of five participating test centers in the canton of Zurich, Switzerland, as part of a diagnostic survey study [64]. The study included five different test sites (four outpatient test centers and one emergency department). Information on symptomatic or asymptomatic status was acquired as part of the regular procedure for SARS-CoV-2 testing and reporting based on self-evaluation (asymptomatic/mild/strong)

by the participants. Viral load in saliva was only measured in this diagnostic cohort and PCR positivity was defined as either NPS or saliva reaching a Ct value < 45. Information on symptomatic or asymptomatic status was acquired as part of the regular procedure for SARS-CoV-2 testing. Reporting was based on self-evaluation (asymptomatic/mild/strong) by the participants [64]. For this study we differentiated between asymptomatic and symptomatic (mild/strong).

4. Saliva sample collection

For saliva collection, individuals of both cohorts were asked to clear their throat thoroughly and collect saliva into a supplied empty tube [64]. As a guidance for the volume of saliva to be sampled, participants were instructed by study teams to collect 0.5–1 mL (approx. a teaspoon full). Immediately after saliva collection, 3 ml of viral transport medium (HEPES (4-(2-hydroxyethyl)-1-piperazineethanesulfonic acid), Dulbecco's Modified Eagle's Medium (DMEM), fetal calf serum (FCS), antibiotics and antimycotics) was added to the collected saliva and the content mixed through gentle tilting.

5. Statistical analysis

Analyses in the present study were designed and conducted retrospectively after the completion of the clinical studies. Summary of the different analyses that were conducted and the corresponding questions that we sought to answer is available in Supplementary Table 1. All statistics obtained for these analyses are summarized in the supplementary material. Statistical analyses were performed in R (Version 4.0.5). Figures were made using the ggplot2 package [109]. When included, boxplots represent the following: median with the middle line, upper and lower quartiles with the box limits, 1.5x interquartile ranges with the whiskers and outliers with points. Significance of Spearman rank correlations were assessed through asymptotic t approximation. Differences in means between two groups with independent measures were tested using two-tailed t-tests. Linear models adjusted for sex and age were used to analyze binding activities (MFI-LFOE). In these models, statistical associations between the outcome and each covariate were assessed using a

Student t-test with a two-sided hypothesis. Tobit-regression models adjusted for sex and age were used to analyze neutralization titers to account for left-censored values, using R package VGAM [110]. Associations between the outcome and covariates were assessed through likelihood-ratio tests. When several linear or tobit-regression models were estimated, we did not correct p-values for multiple testing, as all tested associations were considered because of their a priori biological or clinical plausibility [111]. As the conducted analyses were however not prespecified in a formal analysis plan, results should be interpreted as exploratory. Differences in neutralization activity against the different variants were tested using the BHHH method in the censReg package [112] to account for repeated measurements within individuals and left-censoring. Mediation analysis results were performed in R package mediation [113], and robustness was checked by performing the same analysis in R package mma. Analyses considered symptoms as the outcome, pre-existing HCoV antibody levels as variable of exposure, and SARS-CoV-2 antibody levels upon infection as mediator. Models were adjusted for age, gender, and sub-cohort. We used 500 bootstrap samples in both packages to check for stability of results and compute empirical confidence intervals [114].

2. Supplementary Tables

Supplementary Table 1. Analysis summary.

Summary of the research question		Analysis results	Cohort data included in the analysis		Correspondin g figures
Exposure	Outcome		Plasma	Saliva	
Pre-existing HCoV binding	SARS-CoV-2 binding	Pre-existing HCoV mucosal immunity shapes SARS-CoV-2 antibody response upon infection	Longitudinal (B1-B2, n=85 & C2-C3, n=196)	Longitudinal (B1-B2, n=85)	3A & 3C
SARS-CoV-2 binding	SARS-CoV-2 neutralization	Early induction of SARS-CoV-2 neutralizing activity in saliva	Longitudinal (B2, n=84)		4
SARS-CoV-2 binding	SARS-CoV-2 viral load	Mucosal SARS-CoV-2 antibody response links with viral load	N/A	Cross-sectional (n=177)	5
SARS-CoV-2 binding	SARS-CoV-2 symptoms	Mucosal SARS-CoV-2 antibody response links with lower frequency of symptoms		Cross-sectional (n=155) & longitudinal (B2, n=64)	6A & 6B
Pre-existing HCoV binding	SARS-CoV-2 symptoms	Pre-existing immunity to HCoVs reduces symptomatic SARS-CoV-2 infection	Longitudinal (B1-B2, n=64 & C2-C3, n=151)	Longitudinal (B1-B2, n=58 & C2-C3, n=161)	6C & 6D & 7

Supplementary Table 2. Description of the longitudinal cohort. Samples from children were repeatedly collected during three testing rounds (R1-R3).

	**Round 1 (R1)**<br>**June-July 2020**<br>**(N=2505)**	**Round 2 (R2)**<br>**October-November 2020**<br>**(N=2526)**	**Round 3 (R3)**<br>**March-April 2021**<br>**(N=2455)**	**Overall**<br>**(N=7486)**
**Age**				
Mean (SD)	11.5 (2.52)	11.7 (2.53)	11.9 (2.54)	11.7 (2.54)
Median [Min, Max]	11.6 [6.40, 16.6]	11.8 [6.70, 17.0]	12.0 [7.20, 17.4]	11.8 [6.40, 17.4]
Missing	68 (2.7%)	35 (1.4%)	73 (3.0%)	176 (2.4%)
**Sex**				
Female	1188 (47.4%)	1138 (45.1%)	1118 (45.5%)	3444 (46.0%)
Male	1117 (44.6%)	1060 (42.0%)	1028 (41.9%)	3205 (42.8%)
Other	5 (0.2%)	4 (0.2%)	6 (0.2%)	15 (0.2%)
Missing	195 (7.8%)	324 (12.8%)	303 (12.3%)	822 (11.0%)
**Serostatus**				
Negative	2449 (97.8%)	2387 (94.5%)	2061 (84.0%)	6897 (92.1%)
Positive	56 (2.2%)	139 (5.5%)	394 (16.0%)	589 (7.9%)
**Reported symptoms**				
None	596 (23.8%)	1556 (61.6%)	1494 (60.9%)	3646 (48.7%)
Some	1609 (64.2%)	453 (17.9%)	398 (16.2%)	2460 (32.9%)
Missing	300 (12.0%)	517 (20.5%)	563 (22.9%)	1380 (18.4%)

Supplementary Table 3. Description of the cross-sectional diagnostic cohort.

	**Overall (N=882)**
**Age**	
Mean (SD)	38.7 (17.3)
Median [Min, Max]	35.0 [11.0, 98.0]
**Sex**	
Female	413 (46.8%)
Male	469 (53.2%)
**PCR status**	
Negative	177 (20.1%)
Positive	705 (79.9%)
**Reported symptoms**	
None	228 (25.9%)
Some	621 (70.4%)
Missing	33 (3.7%)

Supplementary Table 4. CoV-derived antigens.

Antigen	Origin	Tag	Expression host	Manufacturer	Catalog number
NP	SARS-CoV-2	C-terminal polyhistidine tag	Baculovirus-Insect cells	Sino Biological Europe GmbH, Eschborn, Germany	50488-V08B
RBD	SARS-CoV-2	C-terminal polyhistidine tag	HEK293 cells		40592-V08H
S1 subunit	SARS-CoV-2	C-terminal polyhistidine tag	HEK293 cells		40591-V08H
S2 subunit	SARS-CoV-2	C-terminal polyhistidine tag	Baculovirus-Insect cells		40590-V08B
S1 subunit	hCoV-HKU1	C-terminal polyhistidine tag	HEK293 cells		40021-V08H
S1 subunit	hCoV-OC43	C-terminal polyhistidine tag	HEK293 cells		40607-V08H1
S1 subunit	hCoV-NL63	C-terminal polyhistidine tag	HEK293 cells		40600-V08H
S1 subunit	hCoV-229E	C-terminal polyhistidine tag	HEK293 cells		40601-V08H

Supplementary Table 5. Origin and characteristics of antibody reagents.

Monclonal / Polyclonal	Isotype	Conjugate	Supplier	Clone	Catalog number	Stock concentration	Dilution in study	Application in study
SARS-CoV-2 (2019-nCoV) Spike S1 monoclonal antibody	Rabbit IgG	-	Sino Biological Europe GmbH, Eschborn, Germany	007	40150-R007	n.a.	1/100	Primary antibody to control antigen coupling to beads
SARS-CoV / SARS-CoV-2 Nucleoprotein / NP polyclonal antibody	Rabbit IgG	-	Sino Biological Europe GmbH, Eschborn, Germany	polyclonal	40143-T62	n.a.	1/100	Primary antibody to control antigen coupling to beads
Anti-His tag monoclonal antibody	Mouse IgG	-	Sino Biological Europe GmbH, Eschborn, Germany	02	105327-MM02T	5 mg/ml		Coupling of His-tagged antigens to beads
Anti-human IgG Fc monoclonal antibody	Mouse IgG	PE	BioLegend, San Diego, CA	HP6017	409304	0.2 mg/ml	1/500	Secondary antibody for ABCORA
Anti-human IgA	Goat IgG	PE	Southern Biotech, Birmingham, AL	polyclonal	2050-09	0.5 mg/ml	1/500	Secondary antibody for ABCORA
Anti-human IgM	Goat IgG	PE	Southern Biotech, Birmingham, AL	polyclonal	2020-09	0.5 mg/ml	1/500	Secondary antibody for ABCORA
Anti-mouse IgG	Goat IgG	PE	BioLegend, San Diego, CA	polyclonal	405307	0.2 mg/ml	1/20	Secondary antibody to control anti-His antibody loading
Anti-rabbit IgG	Goat IgG	PE	Southern Biotech, Birmingham, AL	polyclonal	4030-09	0.5 mg/ml	1/500	Secondary antibody to control antigen coupling

3. Supplementary Figures

Supplementary Figure 1

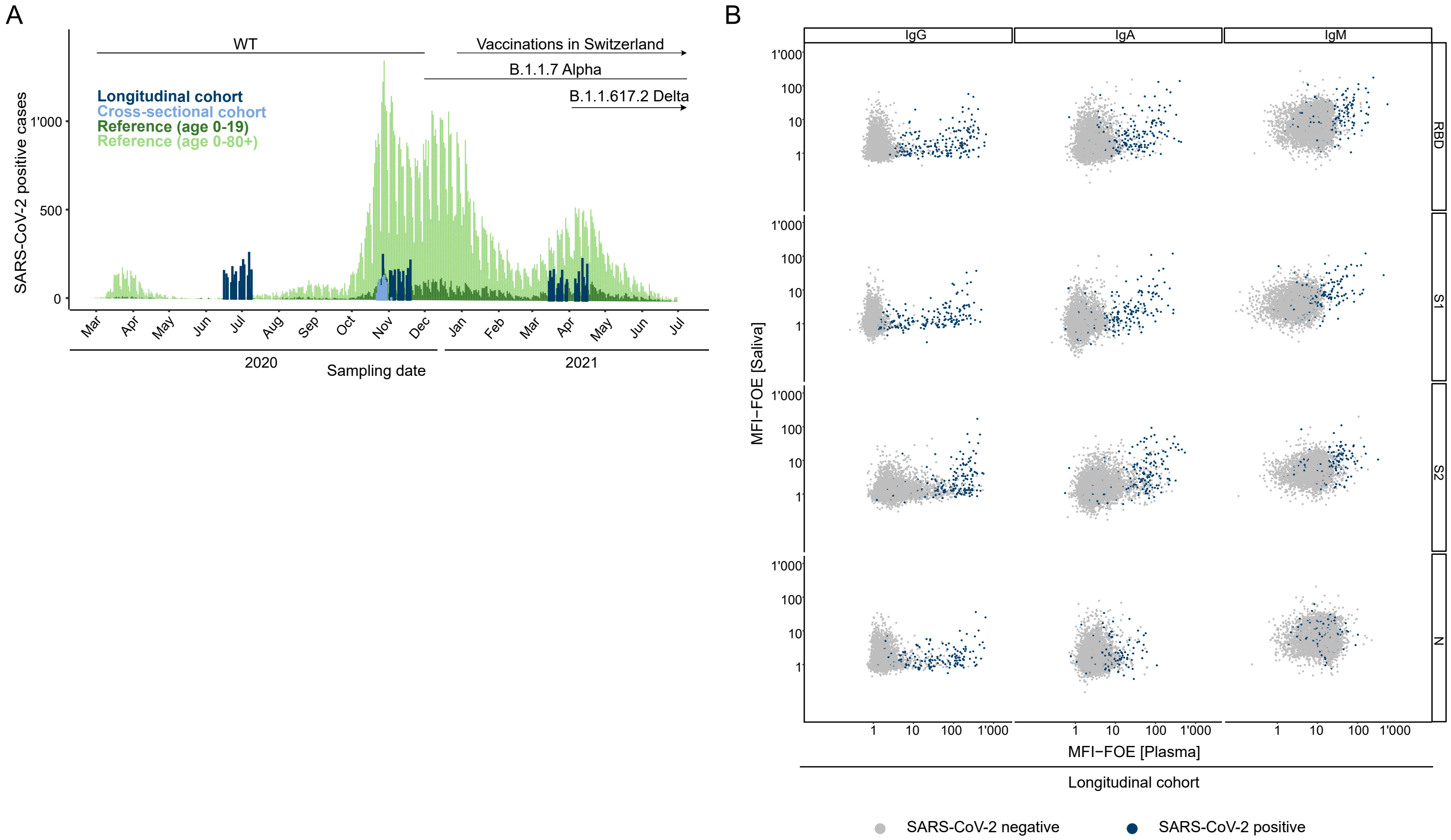

Supplementary Figure 1. A. Sampling dates of the longitudinal (dark blue) and cross-sectional (light blue) cohorts versus reported number of SARS-CoV-2 positives cases in the canton of Zurich, Switzerland, in individuals younger than 20 years-old (dark green) and all individuals (light green). **B.** Antibody reactivities in plasma and saliva in multiplex SARS-CoV-2 ABCORA in plasma in children from the longitudinal cohort with serology measurements at all three visit rounds (n=1967). Depicted are MFI signals normalized for empty bead controls (MFI-FOE). Individuals in light grey stayed seronegative throughout the three visit rounds. SARS-CoV-2 seropositive patients are shown in dark blue.

Supplementary Figure 2

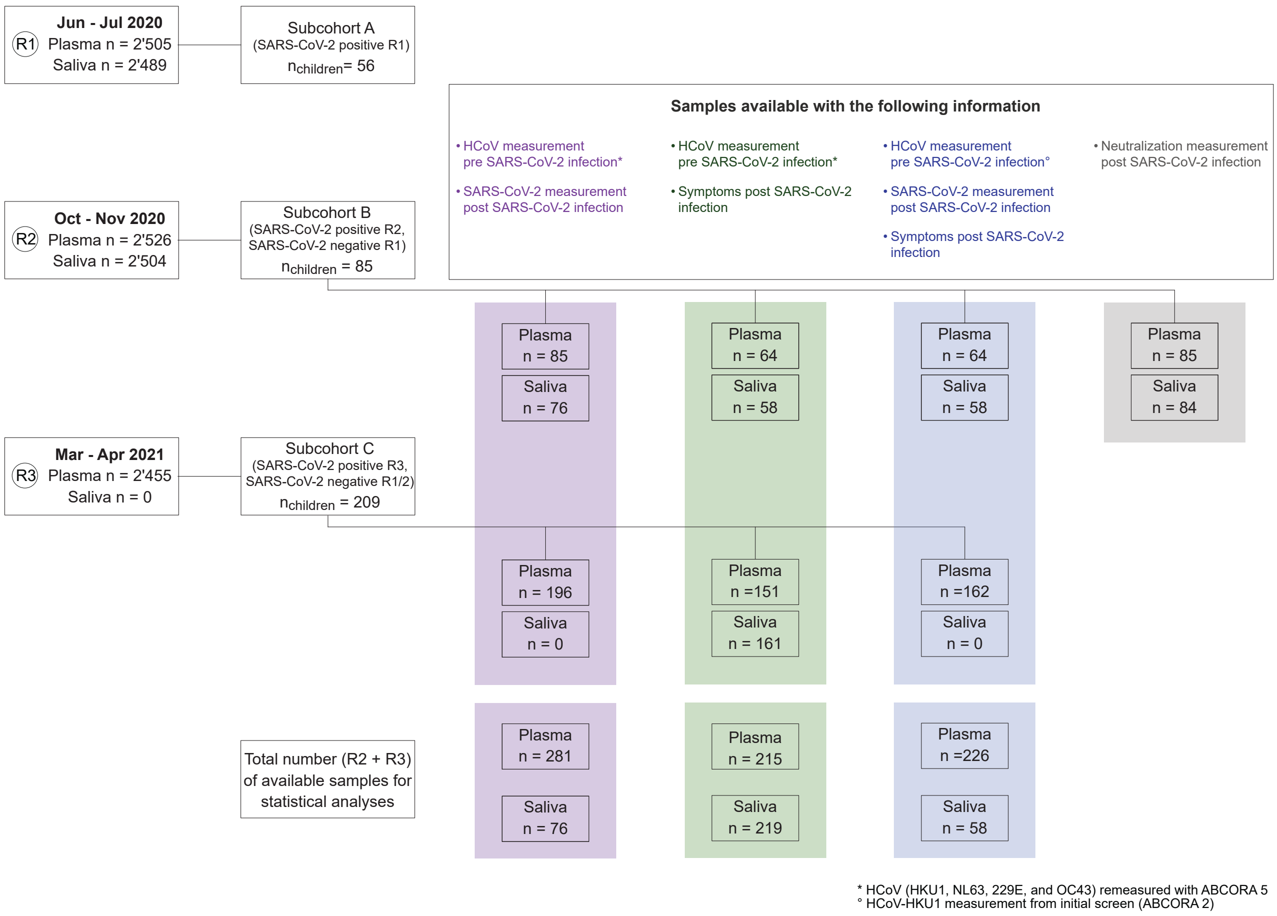

Supplementary Figure 2. Flowchart depicting the selection of the sub-cohorts A, B, and C withing the longitudinal cohort, and the corresponding plasma and saliva samples used in the analyses. At round 3, only plasma samples were collected. Additional measurements were conducted on plasma and saliva samples from children in sub-cohorts B and C only. Number discrepancies in sample size per analysis are explained by the lack of raw material needed for realizing the corresponding measurements. R1=round 1 (June-July 2020), R2=round 2 (October-November 2020), R3=round 3 (March-April 2021).

Supplementary Figure 3

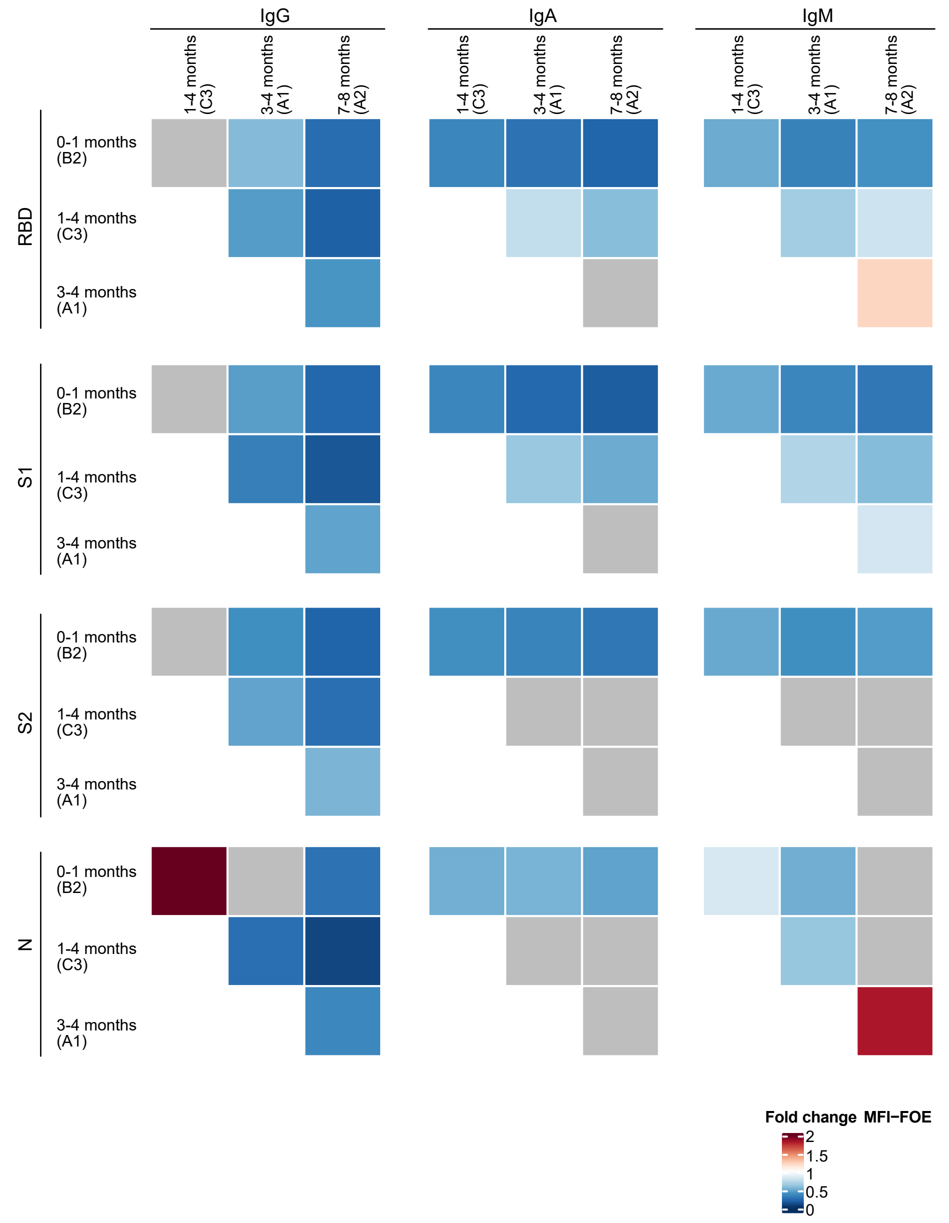

Supplementary Figure 3. Fold-change of all antibody reactivities in plasma between the different sub-cohorts of inferred infection recency (B2: 0-1 months, C3: 1-4 months, A1: 3-4 months, A2: 7-8 months). Differences between all groups are tested using t-tests, except for the difference between A1 and A2 which is tested using a linear mixed effect models with a random effect on the individual (as samples in A1 and A2 consist of the same individuals sampled at different time). Fold changes that are not significant ($p>0.05$) are shown in grey.

Supplementary Figure 4

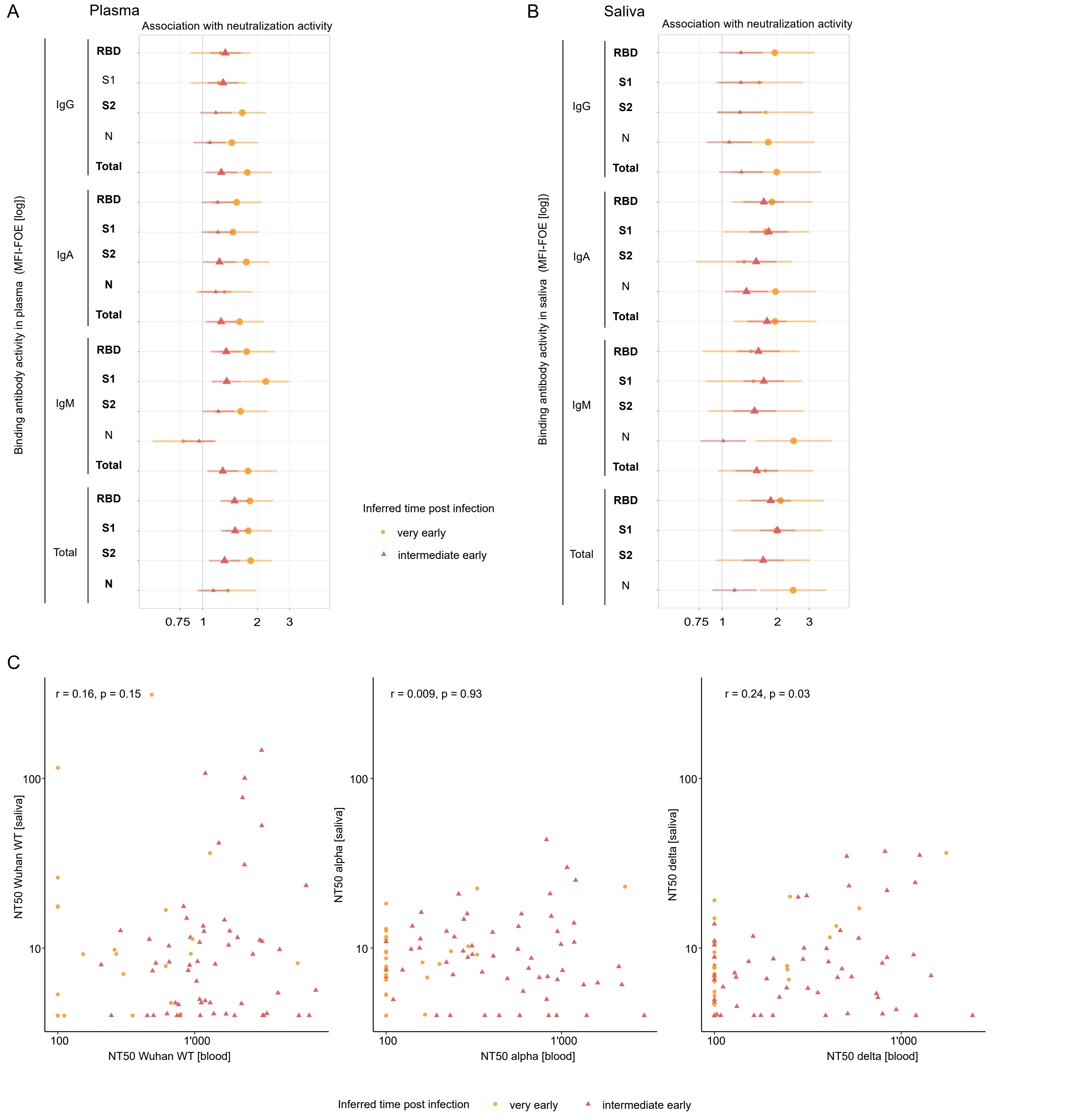

Supplementary Figure 4. A and B. Association coefficients between neutralization titers (NT50) against Wuhan-Hu-1 pseudotype and IgG, IgA, and IgM SARS-CoV-2 antibody titers in plasma (A) and in plasma (B) in individuals very early (orange, n=27) and intermediate early (red, n=58) after infection, obtained with tobit-regression analyses adjusted on age and sex. Binding activities were transformed as time-specific Z-scores (i.e., Z-score were determined in the two different groups: very early and intermediate early after infection). Solid line indicates coefficient estimation for each binding activity in individuals very early (orange, n=27) and intermediate early after infection (red, n=58). Shaded areas correspond to the 95% confidence intervals and grey line corresponds to a null association coefficient. Binding activities significantly associated with neutralization activity ($p<0.05$) are indicated with symbols (very early, circles, intermediate early, triangles). **C.** Correlation between neutralization titers (NT50) against Wuhan-Hu-1 pseudotype (left), Alpha (middle) and Delta (right) in plasma and in saliva with all individuals grouped together (n=85). Individuals from very early (circle, red) and intermediate early (triangle, orange) post infection groups are depicted.

Supplementary Figure 5

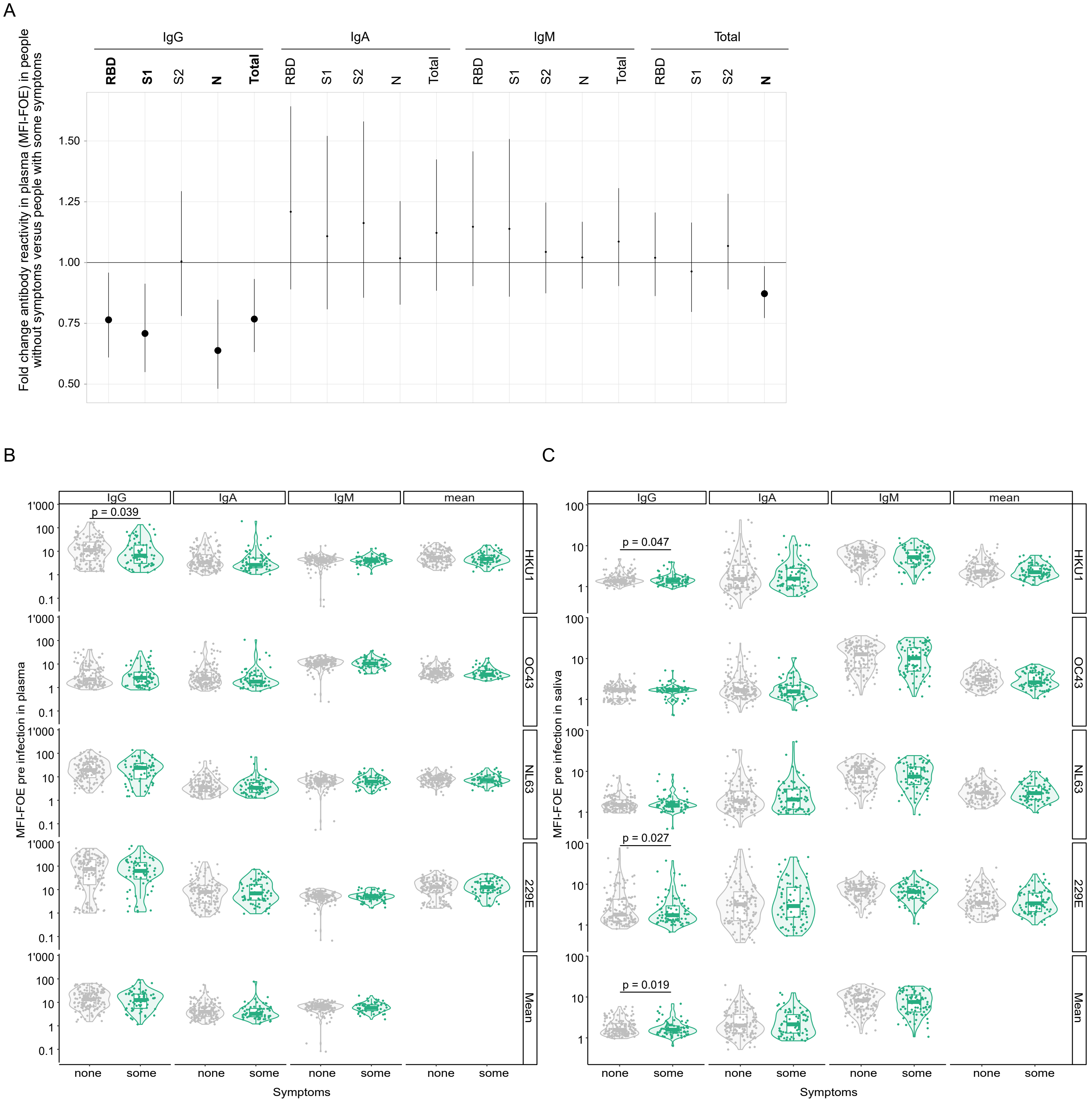

Supplementary Figure 5. A. Linear regression models (n=64) adjusted on age, sex, and inferred recency of infection to assess the fold-change in antibody reactivities in plasma (MFI-FOE) in individuals without symptoms, versus individuals with symptoms. Binding activities with significant difference ($p<0.05$) between those with and without symptoms are indicated in bold. Solid line indicates the fold change obtained from the linear regression model, and shaded areas correspond to the 95% confidence intervals. **B.** Pre-SARS-CoV-2 infection HCoV binding antibodies reactivities in plasma in individuals without symptoms (grey) and with symptoms (green) upon infection. **C.** Pre-SARS-CoV-2 infection HCoV binding antibodies reactivities in saliva in individuals without symptoms (grey) and with symptoms (green) upon infection.
